# Novel IL-15 dendritic cells have a potent immunomodulatory effect in immunotherapy of multiple myeloma

**DOI:** 10.1016/j.tranon.2022.101413

**Published:** 2022-04-09

**Authors:** Tan-Huy Chu, Manh-Cuong Vo, Thangaraj Jaya Lakshmi, Seo-Yeon Ahn, Mihee Kim, Ga-Young Song, Deok-Hwan Yang, Jae-Sook Ahn, Hyeoung-Joon Kim, Sung-Hoon Jung, Je-Jung Lee

**Affiliations:** a BioMedical Sciences Graduate Program, Chonnam National University, South Korea; b Institute of Research and Development, Duy Tan University, Danang, Vietnam; c Department of Hematology-Oncology, Chonnam National University Hwasun Hospital and Chonnam National University Medical School, South Korea

**Keywords:** Immunology, T cells, cytokine induced killer cells, natural killer cells, multiple myeloma

## Abstract

Dendritic cells (DCs) are the most potent antigen-presenting cells, and have thus been used in clinical cancer vaccines. However, the effects of DC vaccines are still limited, leading researchers to explore novel ways to make them effective. In this study, we investigated whether human monocyte-derived DCs generated via the addition of interleukin 15 (IL-15) had a higher capacity to induce antigen-specific T cells compared to conventional DCs. We isolated CD14^+^ monocytes from peripheral blood from multiple myeloma (MM) patients, and induced immature DCs with granulocyte-macrophage colony-stimulating factor (GM-CSF) and IL-4 in the presence or absence of IL-15 for 4–6 days. Then we generated mature DCs (mDCs) with lipopolysaccharide for another 2 days [IL-15 mDCs (6 days), IL-15 mDCs (8 days), and conventional mDCs (8 days)]. IL-15 mDCs (6 days) showed higher expression of MHC I and II, CD40, CD86, and CCR7, and the secretion of IFN-γ was significantly higher compared to conventional mDCs. IL-15 mDCs (6 days) showed superior polarization of naïve T cells toward Th1 cells and a higher proportion of activated T cells, cytokine-induced killer (CIK) cells, and natural killer (NK) cells for inducing strong cytotoxicity against myeloma cells, and lower proportion of regulatory T cells compared to conventional mDCs. These data imply that novel multipotent mDCs generated by the addition of IL-15, which can be cultivated in 6 days, resulted in outstanding activation of T cells, CIK cells and NK cells, and may facilitate cellular immunotherapy for cancer patients.

## Highlights

• Culture DCs with GM-CSF + IL-4 + IL-15 (IL-15 DCs) can be shortened for 6 days.
• IL-15 DCs showed high expression levels of costimulatory receptors, IFN-γ and IL-12p70.
• IL-15 DCs showed strong stimulation toward T, CIK and NK cells.
• The activated lymphocytes showed high cytotoxicity against myeloma cells.

## Introduction

Multiple myeloma (MM) is characterized by the abnormal proliferation of clonal malignant plasma cells and is the second most common cause of hematologic malignancy [1]. Although the development of effective agents has improved survival outcomes for MM patients over the last two decades, MM remains an incurable disease [2, 3]. Recently, cellular immunotherapy has emerged as an important pillar in the treatment of MM, and monoclonal antibodies and chimeric antigen receptor T cells (CAR-T cells) have shown remarkable efficacy in clinical trials with heavily treated MM patients [4, 5]. Dendritic cell (DC)-based vaccines are a promising form of immunotherapy for MM [6].

DCs are the strongest antigen-presenting cells (APCs) that link the innate and adaptive immune systems, thus playing a central role in the initiation of anticancer immunity [6, 7]. Our previous preclinical studies showed that antigen-loaded DC cancer vaccines induced a robust antigen-specific anticancer immune response [3, 8, 9]. However, the clinical efficacy of current DC vaccines is not satisfactory [7, 10]. Therefore, novel ways to develop more effective DC vaccines are highly desirable.

Interleukin 15 (IL-15) is a multifunctional cytokine that is a strong candidate for cancer immunotherapy. IL-15 serves an important role in regulating the activation and function of T cells and natural killer (NK) cells, along with priming the function of components of the innate immune system, such as DCs and macrophages. IL-15 also serves as an instructive cytokine, leading leukocytes to engage in an inflammatory response [11, 12]. Therefore, IL-15 may be an important factor for DC activation and function [11]. Previous studies have reported that IL-15 differentiated bone-marrow-derived DCs into an interferon-producing killer DC (IKDCs)-like phenotype with unique immune characteristics, such as the capacity to secrete IFN-γ [12]. Currently, the most widely accepted protocol for developing DCs for clinical research involves the generation of DCs from peripheral blood monocytes in the presence of granulocyte-macrophage colony-stimulating factor (GM-CSF), IL-4, and maturating agents for 8 days [3, 8, 9, 13]. Previous studies have examined the use of GM-CSF and IL-15, without the inclusion of IL-4 in the generation of DCs, with the highlight on capability to secrete high levels of IFN-γ but low levels of IL-12p70, and reported some immune stimulation to T cells [14, 15]. However, IL-4 is essential in DCs culture and can potently inhibit the macrophages differentiation [16]. Furthermore, a lack of IL-4 may produce immature DCs that are unable to maintain an immature state and thus have decreased antigen-capturing capacity [11, 13]. To overcome these and to further enhance the efficacy of DC vaccines, we used IL-15 with the current protocol for GM-CSF plus IL-4 and applied a short culture duration of 6 days for mature DCs (mDCs), to reduce human resources, expense, and the risk of contamination, which is another critical issue in clinical application.

In this study, we investigated whether generation of monocyte-derived DCs with IL-15 could stimulate immune cells more strongly than conventional DCs. We found that novel multipotent DCs were generated with the addition of IL-15, and that these had high expression of adhesion molecules, costimulatory molecules, and migration receptors, as well as abundant secretion of IFN-γ, indicating outstanding activation of T cells, CIK cells, and NK cells, and thus strong cytotoxicity against target myeloma cells.

## Material and methods

### Ethical approval

Informed consent was obtained from the subjects prior to experiments. All experimental procedures and protocol were approved by the Institutional Review Board of Chonnam National University Hwasun Hospital (IRB No. CNUHH-2019-238).

### Generation of DCs from peripheral blood collected from MM patients

The generation of DCs from peripheral blood collected from MM patients has been described previously [10, 17]. Briefly, peripheral blood mononuclear cells (PBMCs) obtained from peripheral blood of newly diagnosis MM patients, via density-gradient centrifugation with Lymphoprep (Oslo, Norway). Thereafter, monocytes were isolated from PBMC, using the CD14^+^ magnetic activating cell sorting (MACS) system (Miltenyi Biotec Inc, Auburn, CA, USA). The patient's cells expressed positive with HLA-A2 (n = 10) were selected and used in the main study, confirmed via flow cytometry using HLA-A2-FITC (Supplemental Figure 3A). HLA-A2 is the most common MHC class I allele family in humans, and presenting at high frequencies in all populations [18]. Thus we choose to match HLA-A2 in MM patients and cancer cells line (ARH77 and IM9) to mimic an autologous cell-cell interaction, between T cells receptors and MHC class I molecules. Monocytes were then cultured at 5 × 10^5^ cells/well in 6-well plates (BD Falcon, Franklin Lakes, NJ, USA) in RPMI-1640 (Gibco-BRL, Grand Island, NY, USA) supplemented with 10% heat inactivated fetal bovine serum (FBS) (Hyclone, UT, USA) and 1% penicillin-streptomycin (PS) in the presence of 50 ng/mL of recombinant human GM-CSF (rhGM-CSF, Daejon, Korea) with or without 20 ng/mL of recombinant human IL-4 (rhIL-4, R&D System, Minneapolis USA) with or without 10 ng/mL of recombinant human IL-15 (rhIL-15, R&D System). On days 2 and 4, the medium and cytokines were replaced with fresh medium containing cytokines. On days 4 or 6, immature DCs (imDCs) were harvested. Mature DCs (mDCs) were then cultured by a further 48 h of cultivation of imDCs; imDCs were collected, seeded in to 24-well plates (BD Falcon, Franklin Lakes, NJ, USA) in 1ml of RPMI-1640 with 10% FBS and 1% PS, then supplemented with maturating agent, 200 ng/mL of lipopolysaccharide (LPS) from Escherichia coli (Sigma‐Aldrich, St Louis. MO, USA). The maturing DCs were then loaded with 100 Gy gamma-irradiated cancer cell lines, such as ARH77 or IM9 (American Type Culture Collection (ATCC), Rockville, MD, USA), or patient's autologous primary myeloma cells at a ratio of 2:1, 2 h after the addition of maturating agent.

### Morphology of imDCs capture via optical microscope

The CD14+ cells were cultured in 6-well plate (Standard tissue culture treated, Corning, USA). To assess adhesive cells. The morphology of the cells was confirmed on Day 2, 4 and 6; using optical microscope Olympus IX51/TH4-200 system (equipped with camera for taking picture).

### Phenotype analysis

The monoclonal antibodies (mAbs) against human CD83-APC, MHC class I-FITC, MHC class II-FITC, CD40-FITC, CD14-FITC, CD3-FITC, CD80-PE, CD86-PE, CD19-PE, CD11c-PE, CCR7-Pacific blue, and CD56-APC (BD Biosciences, San Jose, CA, USA) were used to detect surface markers on imDCs and mDCs via flow cytometry at Day 6 or Day 8; by staining after washing twice with FACS buffer [phosphate-buffered saline (PBS) containing 10% FBS]. All cells were stained in 100 µL of FACS buffer in 96-well round-bottomed plate (SPL Life Sciences, Korea); then incubated at 4°C for 30 mins in the dark, then washed via centrifugation with FACS buffer and then fixed with 2% paraformaldehyde and stored at 4°C, as described previously [19]. All samples were single color stained. We used isotype controls (IgG1), conjugated with FITC, PE, Pacific Blue, and APC (BD Biosciences). All information about antibodies used for flow cytometry can be found in Table 1. The samples were acquired using FACS Canto (USA), and the data were analyzed using FlowJo (BD Biosciences).

**Table 1:** Antibodies used in Flow cytometry analysis

Antibody	Clone	Company
CD3–FITC	UCHT1	BD Biosciences (San Jose, CA, USA)
CD4-PE	RPA-T4	BD Biosciences (San Jose, CA, USA)
CD4-Vio Blue	REA623	Miltenyi Biotec (CA,USA)
CD8-Vio Green	REA734	Miltenyi Biotec (CA,USA)
CD11c-PE	B-ly6	BD Biosciences (San Jose, CA, USA)
CD14-FITC	M5E2	BD Biosciences (San Jose, CA, USA)
CD19-PE Cy 5	HIB19	BD Biosciences (San Jose, CA, USA)
CD25-PE Cy7	REA570	Miltenyi Biotec (CA,USA)
CD38-PE	HIT2	BD Biosciences (San Jose, CA, USA)
CD40-FITC	5C3	BD Biosciences (San Jose, CA, USA)
CD44-APC Cy7	REA690	Miltenyi Biotec (CA,USA)
CD56-APC	B159	BD Biosciences (San Jose, CA, USA)
CD80-PE	L307.4	BD Biosciences (San Jose, CA, USA)
CD83-APC	HB15	Miltenyi Biotec (CA,USA)
CD86-PE	2331 (FUN-1)	BD Biosciences (San Jose, CA, USA)
CD107a-PE	H4A3	BD Biosciences (San Jose, CA, USA)
CD112-PE	R2.525	Miltenyi Biotec (CA,USA)
CD138-FITC	MI15	BD Biosciences (San Jose, CA, USA)
CD155-PE	PV404.19	Miltenyi Biotec (CA,USA)
MHC I-FITC	G46-2.6	BD Biosciences (San Jose, CA, USA)
MHC II-FITC	G46-6	BD Biosciences (San Jose, CA, USA)
CCR7-Pacific blue	G043H7	BioLegend (CA,USA)
FoxP3-FITC	1054C	R&D Systems (Minneapolis, MN, USA)
Granzyme B-PE	GB11	BD Biosciences (San Jose, CA, USA)
IFN-γ-PE	4S.B3	Invitrogen (Carlsbad, CA, USA)
IL-4-FITC	8D4-8	eBioscience (CA, USA)
NKG2D-PE	1D11	BD Biosciences (San Jose, CA, USA)
MICA-PE	159227	R&D Systems (Minneapolis, MN, USA)
MICB-PE	236511	R&D Systems (Minneapolis, MN, USA)
ULBP-1-PE	170818	R&D Systems (Minneapolis, MN, USA)
ULBP-2/5/6-PE	165903	R&D Systems (Minneapolis, MN, USA)
HLA-E-PE	3D12HLA-E	eBioscience (CA, USA)
PD-L1-APC	MIH1	BD Biosciences (San Jose, CA, USA)
ICAM-1-APC	REA266	Miltenyi Biotec (CA,USA)
HLA-A2-FITC	BB7.2	BD Biosciences (San Jose, CA, USA)
Isotype control (IgG1)-FITC, PE, APC, and Pacific blue	MOPC-21	BD Biosciences (San Jose, CA, USA)
Live/death fixable staining kit-405nm excitation (Amcyan)	Catalog number: L34966	Invitrogen (Carlsbad, CA, USA)

**Dilution:** All reagents has been pre-diluted by manufacturer, and we used at the recommended volume per test following manufacturer instructions. All above antibodies are anti-human antibodies.

### IL-12p70, IL-10, IFN-γ, IL-4, IL-6, and IL-23 production

All imDCs and mDCs were cultured in 96-well plates at 2 × 10^4^ cells/well and stimulated with CD40 ligand (CD40L) transfected J558 cells (as an analog of CD40L expressing T helper cells; a kind gift from Dr. P. Lane, University of Birmingham, United Kingdom) at 5 × 10^4^ cells/well, as described previously [10]. After 24 h, the supernatants were collected, and the production of IL-12p70, IL-10, IFN-γ, IL-4 (ELISA, BD Biosciences), IL-6, and IL-23 (Invitrogen, Carlsbad, CA, USA) was determined using an enzyme-linked immunosorbent assay kit. Each sample was analyzed in triplicate, and the mean absorbance for each set of standards and samples was calculated.

### Phagocytosis assay

ARH77 cancer cells were stained with 0.5 µM of CellTrace CFSE (Invitrogen) at room temperature for 20 min. Then, ARH77 and CFSE-stained ARH77 myeloma cells were gamma-irradiated (100 Gy). Eventually, the gamma-irradiated ARH77 or CFSE-stained ARH77 cancer cells were co-cultured with imDCs at a ratio of 1:2 in the presence of 200 ng/mL LPS for 24 h. After 24 h of co-culture, the mixture of cells was collected and stained with mAbs against human CD11c-PE to detect mDCs; by staining after washing twice with FACS buffer. All cells were stained in 100 µL of FACS buffer in 96-well round-bottomed plate; then incubated at 4°C for 30 mins in the dark, then washed via centrifugation with FACS buffer, the method was described previously [20]. The samples were acquired immediately after CD11c staining, via FACS Canto (USA), and the data were analyzed using FlowJo. The unstained (mDCs, ARH77 or mDCs-ARH77 co-cultured) or Fluorescence Minus One (FMO) controls were used as appropriate.

### Polarization of naive allogeneic CD4^+^ T cells

The maturing DCs were loaded with 100 Gy gamma-irradiated cancer cell lines (ARH77 or IM9). Then, we co-cultured those matured DCs (5 × 10^4^ cells) with allogeneic CD4^+^ T cells (5 × 10^4^ cells) that were isolated from other PBMCs from MM patients using the CD4^+^ MACS system (Miltenyi Biotec Inc, Auburn, CA, USA), in RPMI-1640 medium containing 10% FBS and 1% PS. On day 3, 5 ng/mL of recombinant human IL-2 (rhIL-2, R&D System, USA) was added. Then, the medium and cytokines were replaced every 2 days with fresh medium containing cytokines. On day 7, the T cells were harvested and re-stimulated with 1 µg/mL of CD3/CD28 mAbs for 24 h. Then we harvested the cells and assessed intracellular production of IL-4 and IFN-γ, as well as the population of regulatory T cells. To measure IL-4 and IFN-γ production, we added a protein transport inhibitor containing Brefeldin (BD Golgi Plug^TM^) at 1 µL/10^6^ cells during the final 5 h of re-stimulation time. We then used the mAbs against a LIVE/DEAD Fixable Aqua Dead Cell stain kit (405 nm excitation-525 nm emission - Amcyan), human CD-4-PE; by staining after washing twice with FACS buffer. All cells were stained in 100 µL of FACS buffer in 96-well round-bottomed plate; then incubated at 4°C for 30 mins in the dark, then washed via centrifugation with FACS buffer. Then subsequently we conducted intracellular staining with mAbs against IL-4-FITC, and IFN-γ-APC to detect intracellular markers of the cell mixture. To detect regulatory T cells, we used the mAbs against human CD25-PE-Cy7 and CD4-PE for surface staining, and subsequently conducted intracellular staining with mAbs against FoxP3-FITC. These intracellular staining steps followed the FACs permeable solution (BD Biosciences) protocol. All information about antibodies used for flow cytometry can be found in Table 1. The samples were acquired immediately after intracellular staining, using FACs Canto (USA), and the data were analyzed using FlowJo. The unstained or FMO controls were used as appropriate.

### Induction of myeloma-specific T cells and other lymphocytes

The maturing DCs (1 × 10^5^ cells) were loaded with 100 Gy gamma-irradiated cancer cell lines (ARH77 or IM9) or primary autologous myeloma cells, were co-cultured with 1 × 10^6^ autologous PBMCs obtained from the same patients. On day 3, we added 5 ng/mL of rhIL-2 and 10 ng/mL of recombinant human IL-7 (rhIL-7, R&D System, USA). Every 2 days, the medium and cytokines were replaced with fresh medium containing cytokines. The autologous T cells were re-stimulated with the same cryo-preserved mDCs on day 7. Finally, on day 12, the cell mixture was collected and the number of antigen-specific T cells and the activation of CIK cells and NK cells were analyzed via the IFN-γ enzyme-linked immunospot (ELIspot) assay (BD Biosciences). The indirect cytotoxic assay based on the IFN-γ ELIspot assay was described previously [17]. The frequency of antigen-specific T cells was analyzed using ARH77 or IM9 cancer cells at specific target cells. Specifically, NK cells were analyzed using K562 cancer cells, and CIK cells were analyzed using ARH77, IM9, and K562 cancer cells (ATCC, USA). The ELIspot data were expressed as the mean number of spots per 1 × 10^5^ lymphocytes. Lymphocytes alone were used as the background control group. The mixture of lymphocytes was stained with mAbs against a LIVE/DEAD Fixable Aqua Dead Cell stain kit, human CD8-Pacific Blue (Vio Blue), CD8-Amcyan (Vio Green), CD3-FITC, CD4-PE, CD56-APC, CD44-APC-Cy7, CD25-PE-Cy7, NKG2D-PE; by staining after washing twice with FACS buffer. All cells were stained in 100 µL of FACS buffer in 96-well round-bottomed plate; then incubated at 4°C for 30 mins in the dark, then washed via centrifugation with FACS buffer. Then subsequently we conducted intracellular staining with mAbs against FoxP3-FITC, IL-4-FITC, Granzyme B-PE and IFN-γ-PE (Miltenyi Biotec Inc, USA) to detect effector CD4^+^ T cells, CD8^+^ T cells, CIK cells, NK cells, and regulatory T cells. These intracellular staining steps followed the FACs permeable solution (BD Biosciences) protocol. The samples were acquired immediately after intracellular staining, using FACS Canto (USA), and the data were analyzed using FlowJo. The unstained or FMO controls were used as appropriate.

### CD107a expression in lymphocytes

The mixture of effector autologous lymphocytes (5 × 10^4^ cells) was stained with anti-human CD107a-PE (BD Biosciences, USA) and incubated with ARH77, IM9, K562, and patient autologous primary myeloma cells with an effector/target ratio of 1:1, in 200µL of RPMI-1640 with 10% FBS and 1% PS, in 96-well plates for 4 h at 37°C with 5% CO2. After 4 h of incubation, the cells were stained with mAbs against a LIVE/DEAD Fixable Aqua Dead Cell stain kit, anti-human CD56-APC, and CD3-FITC; by staining after washing with FACS buffer. All cells were stained in 100 µL of FACS buffer in 96-well round-bottomed plate; then incubated at 4°C for 30 mins in the dark, then washed via centrifugation with FACS buffer; to determine the populations of T cells, CIK cells, and NK cells. The samples were acquired immediately after surface staining, using FACS Canto (USA), and the data were analyzed using FlowJo. The unstained or FMO controls were used as appropriate.

### Cancer cells line culture

ARH77 (plasma cell leukemia), IM9 (multiple myeloma) and K562 (chronic myelogenous leukemia) cancer cells (ATCC, Rockville, MD, USA), were cultured in RPMI 1640 medium (10% FBS, 1% PS) in a 100 × 20 mm cell culture dish (Corning, USA).

### Collection of CD138^+^ primary myeloma cells from the bone marrow of MM patients

Mononuclear cells were obtained from the bone marrow of newly diagnosed MM patients (n = 12), immediately after bone marrow aspiration. Next, myeloma cells were isolated using the CD138^+^ MACS system (Miltenyi Biotec Inc, Auburn, CA, USA). Isolated myeloma cells were then cryopreserved, enabling them to be used as an irradiated antigen-source for DCs, and as autologous primary myeloma cells. Isolated primary myeloma cells also were stained with mAbs against human CD38-PE, CD138-FITC, CD19-PE-Cy5.5, MHC class I-FITC, MICA-PE, MICB-PE, ULBP1-PE, ULBP-2/5/6-PE, HLA-E-PE, PD-L1-APC, CD112-PE, CD155-PE, and ICAM-1-APC (R&D System, USA) to determine morphology, the surface staining steps were described above. The samples were acquired using FACS Canto (USA), and the data were analyzed using FlowJo.

### Lactate dehydrogenase (LDH) release cytotoxic assay

The quantitative cytotoxic assay for measuring LDH was as described previously [21]. We used the CytoTox 96 non-radioactive cytotoxicity assay (Promega, USA) to analyze the direct killing effect of activated lymphocytes against patient autologous primary myeloma cells, according to the manufacturer's instructions. The patients’ autologous primary myeloma cells were pre-stained without or with 2 µg of anti-human HLA-A, B, or C antibody (clone: W6/32, Biolegend, USA) per 10^6^ cells in 100 µL of RPMI media for 20 min, and then washed via centrifugation. The patient primary myeloma cells were co-cultured with activated lymphocytes at a 1:2 ratio in a Costar 96-well plate (Corning, USA) for 6 h at 37°C with 5% CO_2_. Then the supernatants were collected to measure the concentration of LDH, which is the cytosolic enzyme that is released upon cell lysis. The percentage of cell lysis was calculated according to the manufacturer's instructions.

### Genomic Data Commons (GDC) Data analysis

Multiple Myeloma CoMMpass Study data from GDC were used and analyzed using Xenabrowser.net web server for comprehensive analysis [22]. The analysis was performed according to the web server instruction, in March 2021.

### Statistical analysis

All statistical analyses were performed using GraphPad Prism 8 for Windows 10 (Microsoft, USA). T-tests and one-way ANOVA tests were used as appropriate. A P value of < 0.05 was considered significant. Values are expressed as mean ± SDs.

## Results

### The important of IL-4 in ex vivo DCs culture

**Figure 1:**
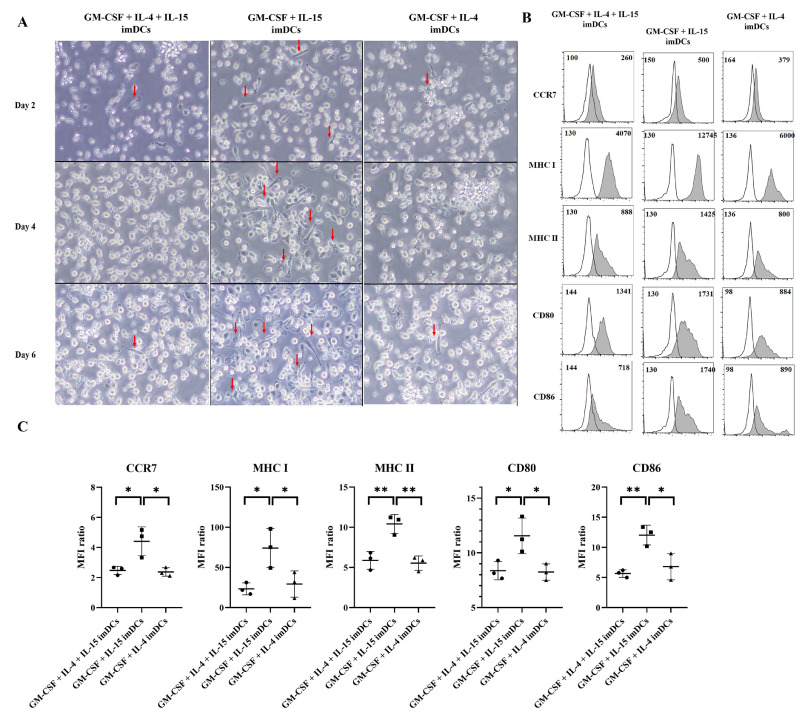
**IL-4 is essential for ex vivo dendritic cells (DCs) culture.** We established three group of DCs culture. GM-CSF + IL-4 + IL-15, GM-CSF + IL-15, and GM-CSF + IL-4. **(A)** The morphology of immature (im) DCs was investigated by optical microscope Olympus IX51/TH4-200 system on Day 2, 4 and 6 of culture process. The pictures showed that on Day 2, 4 and 6 there was an excessive growth of adhesive cells on GM-CSF + IL-15 imDCs group (red arrow), which indicate for the growth macrophages and mature DCs. Next, we collected the imDCs at Day 6, and investigate for the expression of DCs mature marker, such as CCR7, MHC I and II, CD80, CD86 by flow cytometry. The data was showed **(B)** via representative histogram (sample in shaded, compared to isotype control in black line) and **(C)** via MFI ratio (MFI of sample/MFI of isotype control) graphs. The flowcytometry data showed that the lack of IL-4 in GM-CSF + IL-15 imDCs group, make DCs unable to maintain immature state, which represent in increasing expression levels of CCR7, MHC I and II, CD80, CD86, and thus have decreased antigen-capturing capacity. Data are representative from three independent experiments (n =3). *P < 0.05; ** P < 0.01 (One-way ANOVA, multiple comparisons test: Tukey).

Firstly, in the screening study, we established three experimental groups: GM-CSF + IL-4 + IL-15 imDCs (our target), GM-CSF + IL-15 imDCs (in previous studies), and GM-CSF + IL-4 imDCs (conventional); imDCs were culture for 6 days. The morphology of imDCs was investigated by optical microscope on Day 2,4 and 6 of culture process. Data showed an excessive growth of adhesive cells in GM-CSF + IL-15 imDCs group, which indicate for the growth of macrophages and mature DCs (Figure 1A). Next, we collected the imDCs at Day 6, and investigate the expression of DCs marker by flow cytometry. The data showed that the lack of IL-4 in GM-CSF + IL-15 imDCs group make DCs unable to maintain immature state, which represent in significant higher levels of CCR7, MHC I and II, CD80, CD86 compared to other groups (Figure 1B and 1C), thus decreased antigen capture ability**.** At Day 6, imDCs were matured by addition of LPS and loaded with ARH77 irradiated cancer cells, for another 2 days. At Day 8, all imDCs (serve as negative control) and mDCs groups were collected and their surface markers were investigated by flowcytometry. All mDCs groups showed increased expression levels of CCR7, MHC I and II, CD80, CD86 compared to their imDCs counterparts. However, GM-CSF + IL-15 mDCs group did not showed significant different in expression of CCR7 migration receptor compared to GM-CSF + IL-15 imDCs. GM-CSF + IL-4 + IL-15 mDCs group showed significant increase in expression of CD80 and CD86 compared to GM-CSF + IL-15 mDCs group and GM-CSF + IL-4 mDCs group (Supplemental Figure 1A and 1B). Additionally, on day 4 of culture, GM-CSF + IL-4 + IL-15 imDCs group showed a high percentage of CD11c+ cells (>93%) whereas the proportion of CD14+ cells was < 1% on day 4 of culture (Supplementary Figure 1C and 1D). Furthermore, GM-CSF + IL-4 + IL-15 mDCs group secrete significantly higher level of IFN-γ and IL-12p70 compared to GM-CSF + IL-15 mDCs group (Supplemental Figure 2A and 2D); and GM-CSF + IL-15 mDCs group did not showed significant different in secretion levels of IL-12p70 compared to GM-CSF + IL-15 imDCs group (Supplemental Figure 2D). The production of IL-12p70 is the best understood DC-derived cytokine that involve in T_H_1 polarization. These results indicated that IL-4 is essential in ex vivo DCs culture, and our targeted GM-CSF + IL-4 + IL-15 DCs group are more sufficient than GM-CSF + IL-15 DCs.

### Characteristics of immature and mature DCs

**Figure 2:**
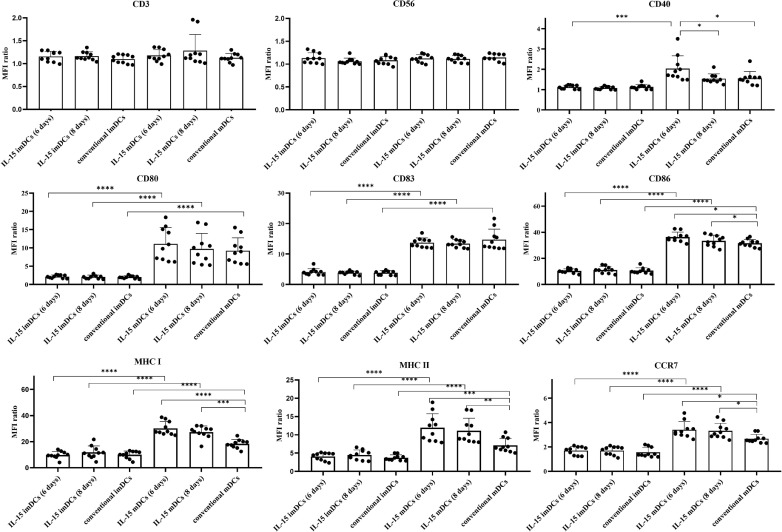
**Characterization of dendritic cells (DCs).** We analyzed the DC phenotype for expression levels of CD3, CD56, CD40, CD80, CD83, CD86, CCR7, MHC I, and MHC II using flow cytometry. The MFI ratios (MFI of samples/MFI of isotype controls) of each sample are shown as bar graphs. All of the mature DCs (mDCs) groups showed increased expression of maturation markers (CD80, CD83, CD86) compared to the immature DCs (imDCs) groups. The IL-15 mDCs (6 days) group showed significant increased expression of MHC I, MHC II, CD40, CD86, and CCR7 compared to the conventional mDCs group. Data are representative from ten independent experiments (n = 10). *P < 0.05; ** P < 0.01; *** P < 0.001; **** P < 0.0001 (One-way ANOVA, multiple comparisons test: Tukey).

**Figure 3:**
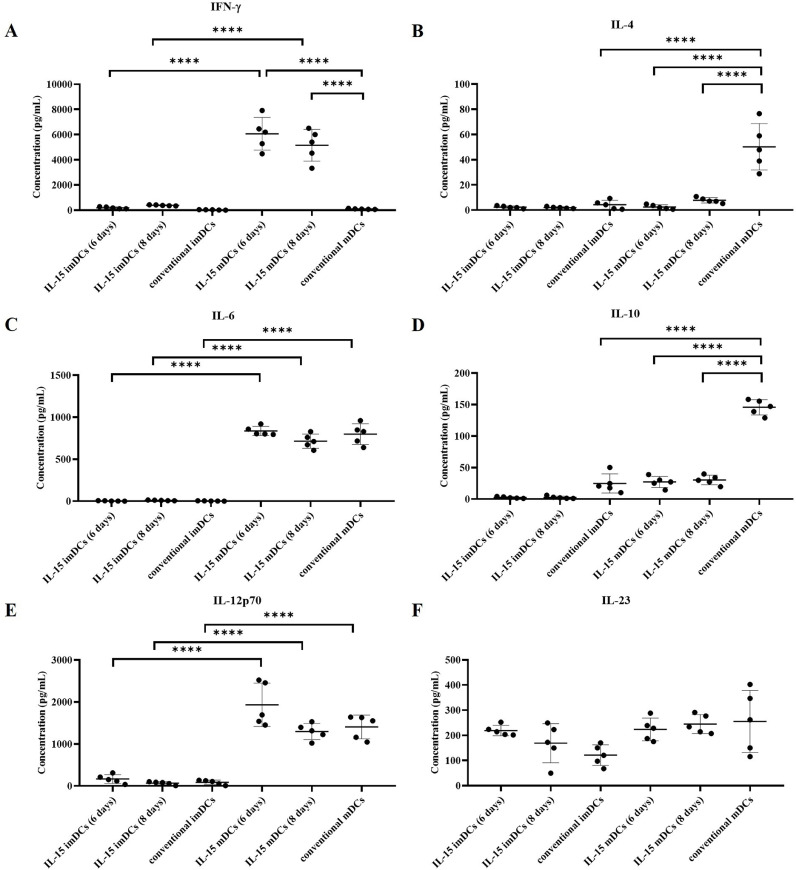
**IL-15 mDCs produced cytokines toward the enhanced activation of immune system.** ELISA results from supernatants of DCs cultured with CD40L-transfected J558 cells show that the IL-15 mDCs (6 days) and IL-15 mDCs (8 days) groups produced higher level of IFN-γ and lower levels of IL-4 and IL-10 compared to the conventional mDCs group. Levels of IL-6, IL-12p70, and IL-23 did not differ among the mDCs groups. Data are representative from five independent experiments (n =5). *P < 0.05; ** P < 0.01; *** P < 0.001; **** P < 0.0001; ns: no significant difference (One-way ANOVA, multiple comparisons test: Tukey).

Secondly, in the main study, three experimental groups of mDCs were established: 1) GM-CSF + IL-4 + IL-15 for 4 days and lipopolysaccharide (LPS) for 2 days [IL-15 mDCs (6 days)], 2) GM-CSF + IL-4 + IL-15 for 6 days and LPS for 2 days [IL-15 mDCs (8 days)], and 3) GM-CSF + IL-4 for 6 days and LPS for 2 days (conventional mDCs) as a control group. To generate mDCs, imDCs on day 4 or day 6 were stimulated by LPS for further 2 days. Gamma-irradiated ARH77, IM9, or patient primary myeloma cells were added 2 h after the addition of LPS. The mDCs from all three groups showed significantly increased expression of the maturation markers CD80, CD83, CD86, and CCR7 compared to imDCs (Figure 2 and Supplemental Figure 4). In addition, the IL-15 mDCs (6 days) group showed significantly increased expression of CD40, CD86, CCR7, MHC I, and MHC II compared to the conventional mDCs group (Figure 2 and Supplemental Figure 4). There were no significant differences in the expression of CD3 or CD56 between the imDCs and mDCs groups (Figure 2). In addition, the IL-15 mDCs (6 days) and IL-15 mDCs (8 days) groups secreted very high levels of IFN- γ (Figure 3A). The IL-15 mDCs (6 days) and IL-15 mDCs (8 days) groups showed significantly decreased production of IL-4 and IL-10 compared to the conventional mDCs group (Figure 3B and 3D). However, levels of IL-6 and IL-12p70 production did not differ significantly between the mDCs groups (Figure 3C and 3E), and IL-23 production did not differ between the immature and mature DCs groups (Figure 3F). The phagocytosis assay also confirmed the mDCs antigen uptake capacity against irradiated ARH77 cancer cells (Supplemental Figure 3C). These results indicate that the DCs generated with IL-15, especially those in the IL-15 mDCs (6 days) group, exhibited high expression of adhesion molecules, costimulatory molecules, and migration markers, produced lower levels of IL-4 and IL-10, and had very high levels of activated cytokines (IFN-γ), which could further boost the activation of immune system.

### Enhancement of allogeneic Th1 polarization and suppression of allogeneic regulatory T cells by DCs generated with IL-15

**Figure 4:**
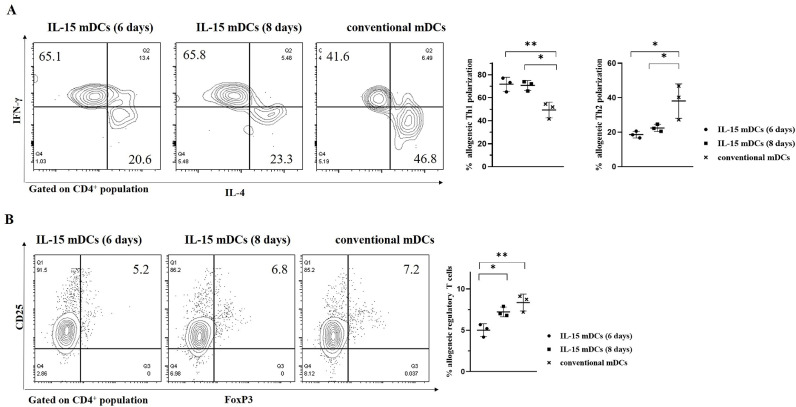
**IL-15 mDCs have an immunomodulatory function to induce allogeneic Th1 polarization and to suppress allogeneic regulatory T cells. (A)** The intracellular staining of IFN-γ and IL-4 on isolated allogeneic CD4^+^ T cells was measured using flow cytometry. The IL-15 mDCs (6 days) group and IL-15 mDCs (8 days) group showed stronger polarization toward Th1 cells and lower polarization toward Th2 cells compared to the conventional mDCs group. **(B)** The proportion of regulatory T cells was decreased in the IL-15 mDCs (6 days) group compared to the conventional mDCs group. Data are representative from three independent experiments (n = 3). *P < 0.05; ** P < 0.01 (One-way ANOVA, multiple comparisons test: Tukey).

To investigate the polarization of T cells activated by mDCs, we evaluated the polarization capacity of isolated CD4^+^ naïve allogeneic T cells when stimulated by mDCs. Allogeneic CD4^+^ T cells stimulated with cells from the IL-15 mDCs (6 days) and IL-15 mDCs (8 days) groups showed significantly increased production of IFN-γ and decreased production of IL-4 compared to the conventional mDCs group (Figure 4A). In addition, inhibition of allogeneic regulatory T cells (CD4^+^CD25^+^FOXP3^+^) was stronger in the IL-15 mDC (6 days) group compared to the other groups (Figure 4B). These results indicate that DCs generated with IL-15, especially those in the IL-15 mDCs (6 days) group, had a stronger immunomodulatory effect that could promote the Th1 polarization of allogeneic naïve CD4^+^ T cells and indirectly suppress regulatory T cells via mDCs in MM patients.

### Enhancement of autologous CD4^+^ T cell, CD8^+^ T cell, CIK cell, and NK cell activation and suppression of autologous regulatory T cells by DCs generated with IL-15

**Figure 5:**
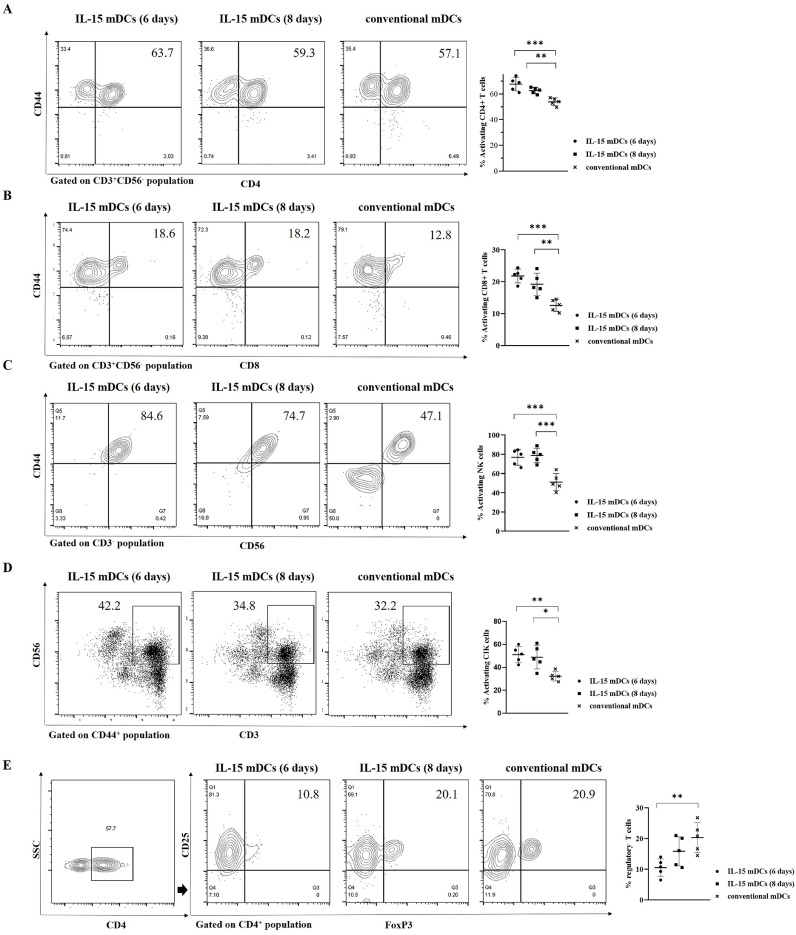
**IL-15 mDCs enhanced the activation of autologous T cells, CIK cells, and NK cells, and the suppression of autologous regulatory T cells.** The proportions of activated CD4^+^ T cells, CD8^+^ T cells, NK cells, CIK cells, and regulatory T cells were measured via flow cytometry. The IL-15 mDCs (6 days) group showed a significant increase in the proportions of activated CD4^+^ T cells **(A)**, CD8^+^ T cells **(B)**, NK cells **(C)**, and CIK cells **(D)**, and a decrease in the proportion of regulatory T cells **(E)** compared to the conventional mDCs group. Data are representative from five independent experiments (n = 5). *P < 0.05; ** P < 0.01; *** P < 0.001 (One-way ANOVA, multiple comparisons test: Tukey).

To investigate the activation of autologous lymphocytes by mDCs, we evaluated the activation of CD4^+^ T cells, CD8^+^ T cells, CIK cells, and NK cells following stimulation via mDCs. The flow cytometry gating strategies for autologous lymphocytes are described (Supplemental Figure 5). Autologous CD4^+^ T cells stimulated by mDCs from the IL-15 mDCs (6 days) group and IL-15 mDCs (8 days) group showed a significantly increased proportion of activated CD3^+^CD56^−^CD4^+^CD44^+^ T cells and enhanced production of IFN-γ compared to conventional mDCs group (Figure 5A and Supplemental Figure 6A). Autologous CD8^+^ T cells stimulated by mDCs from the IL-15 mDCs (6 days) group and the IL-15 mDCs (8 days) group showed a significantly increased proportion of activated CD3^+^CD56^−^CD8^+^CD44^+^ T cells and enhanced production of IFN-γ compared to the conventional mDCs group (Figure 5B and Supplemental Figure 6B), although no significant differences were found in intracellular granzyme B expression in CD8^+^ T cells between the groups (Supplemental Figure 7A). NK cells stimulated by mDCs from the IL-15 mDCs (6 days) group and the IL-15 mDCs (8 days) group showed an increased proportion of activated CD3^−^CD56^+^CD44^+^ NK cells and enhanced production of IFN-γ, as well as increased expression of NKG2D compared to the conventional mDCs group. However, only IL-15 mDCs (6 days) group showed increased in expression of granzyme B compared to the other groups (Figure 5C, Supplemental Figures 6D, 7B, and 8A). Notably, CIK cells stimulated by IL-15 mDCs (6 days) showed an increased proportion of activated CD3^+^CD56^+^CD44^+^ CIK cells and enhanced production of IFN-γ, as well as increased expression of granzyme B and NKG2D compared to the other groups. However, there is no significant difference in the proportion of CD3^+^CD56^+^CD44^+^ CIK cells and the expression of NKG2D between IL-15 mDCs (6 days) and IL-15 mDCs (8 days) groups (Figure 5D, Supplemental Figures 6C, 7C, and 8B). The representative population of each activated cell type in lymphocytes was described via t-distributed stochastic neighbor embedding (tSNE) (Supplemental Figure 9). In addition, the inhibition of autologous regulatory T cells was stronger in the IL-15 mDCs (6 days) group compared to the conventional mDC groups (Figure 5E). These results indicate that DCs generated with IL-15, especially those in the IL-15 mDCs (6 days) group, enhanced the activation and function of autologous CD4^+^ T cells, CD8^+^ T cells, CIK cells, and NK cells, and also suppressed regulatory T cells, indicating to have a multipotent immunomodulatory role.

### Robust enhancement in cytotoxicity of autologous T cells, CIK cells, and NK cells by DCs generated with IL-15

**Figure 6:**
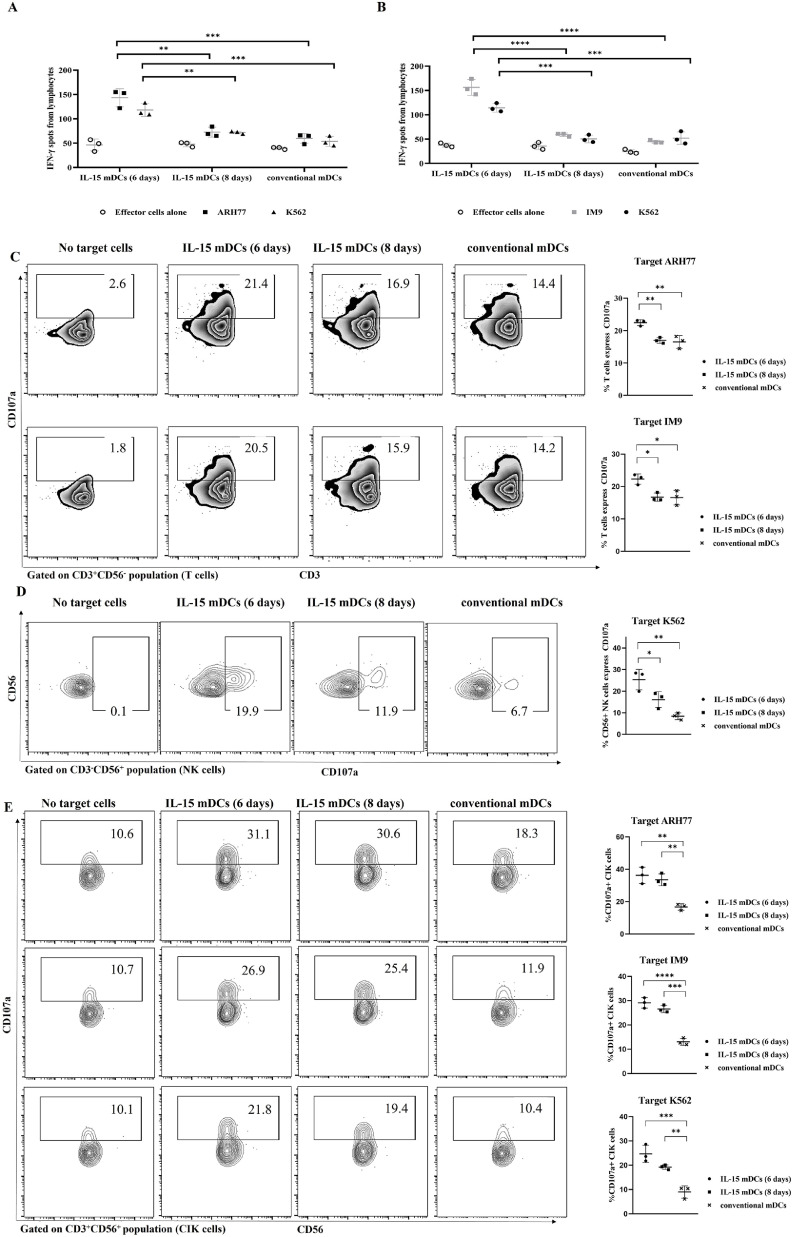
**IL-15 mDCs induced robust enhancement of cytotoxicity by generation of potent autologous cytotoxic T lymphocytes, CIK cells, and NK cells.** An IFN-γ ELIspot assay was performed to quantify IFN-γ production of the activated lymphocytes by mDCs against **(A)** ARH77 or **(B)** IM9. The activated cells by IL-15 mDCs (6 days) group showed significantly increased the numbers of IFN-secreting spots against ARH77, IM9, and K562 target cells compared to those by conventional mDCs. We measured the expression of degranulation marker CD107a via flow cytometry to evaluate the cytotoxicity of **(C)** T cells against ARH77 and IM9, **(D)** NK cells against K562, and **(E)** CIK cells against ARH77, IM9, and K562. The IL-15 mDCs (6 days) group exhibited significantly increased expression of CD107a in all of the generated cells compared to those of conventional mDC group. These data indicate that CTLs, CIK cells, and NK cells generated by IL-15 mDCs (6 days) group had stronger cytotoxicity than those of the conventional mDCs group. Data are representative from three independent experiments (n = 3). *P < 0.05; ** P < 0.01; *** P < 0.001; **** P < 0.0001 (Multiple t tests – one per row for A, B. One-way ANOVA, multiple comparisons test: Tukey for C, D and E).

To investigate the anti-myeloma effect of specific T cells activated by mDCs loaded with gamma-irradiated cancer cells (ARH77 or IM9), we performed an indirect cytotoxic IFN-γ ELIspot assay to track IFN-γ production by T cells, CIK cells, and NK cells, along with a CD107a degranulation assay. ARH77 and IM9 cancer cells express HLA-A2, which are the target cells for T cells and CIK cells, and K562 cancer cells do not express HLA-A2 (Supplemental Figure 3B), which is the target cells for NK cells and CIK cells. Anti-ARH77 T cells and CIK cells stimulated with IL-15 mDCs (6 days) displayed higher IFN-γ spot production against ARH77 cancer cells compared with the other groups (Figure 6A). Similarly, anti-IM9 T cells and CIK cells stimulated with IL-15 mDCs (6 days) displayed higher IFN-γ spot production against IM9 cancer cells compared to the other groups (Figure 6B). CIK cells and NK cells stimulated with IL-15 mDCs (6 days) in two separate experiments also displayed more IFN-γ production spots against K562 cells than the other groups (Figure 6A and 6B). In addition, we investigated the expression of degranulation marker CD107a in CD3^+^CD56^−^ specific anticancer T cells when co-cultured with ARH77 or IM9 cancer cells. T cells stimulated with IL-15 mDCs (6 days) showed the highest expression of CD107a compared to the other groups (Figure 6C). Next, we investigated the expression of degranulation marker CD107a in CD3^−^CD56^+^ NK cells when co-cultured with K562 cancer cells. Notably, NK cells stimulated with IL-15 mDCs (6 days) showed significantly increased expression of CD107a compared to the conventional mDC group (Figure 6D). Lastly, we investigated the expression of degranulation marker CD107a in CD3^+^CD56^+^ CIK cells against ARH77, IM9, and K562 cancer cells. CIK from the IL-15 mDCs (6 days) group and IL-15 mDCs (8 days) group showed significantly increased expression of CD107a compared to the conventional mDCs group (Figure 6E). “No target cells” group serve as negative control, represent for effector cells alone. These results indicate that DCs generated with IL-15, especially those in the IL-15 mDCs (6 days) group, can robustly enhance the cytotoxicity and function of specific anti-cancer T cells, CIK cells, and NK cells, as indicated by the numbers of IFN-γ spots and levels of CD107a expression against ARH77, IM9, and K562 target cells.

### Robust enhancement of cytotoxicity of lymphocytes stimulated by DCs loaded with IL-15 against autologous primary myeloma cells (paired)

**Figure 7:**
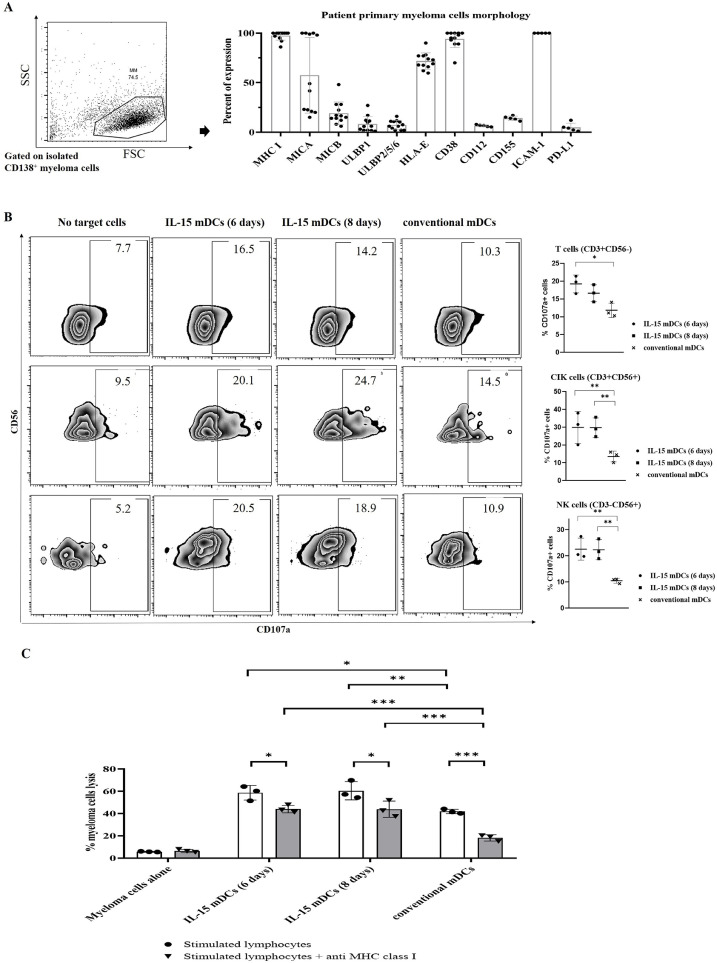
**IL-15 mDCs showed the enhanced cytotoxicity by generation of potent autologous cytotoxic T lymphocytes, CIK cells, and NK cells against primary autologous myeloma cells (paired). (A)** We analyzed CD138^+^ primary myeloma cells isolated from bone marrow aspiration samples of newly diagnosed multiple myeloma patients to assess the expression levels of MHC class I, MICA, MICB, ULBP1, ULBP2/5/6, HLA-E, CD38 (n = 12), and CD112, CD155, ICAM-1 and PD-L1 (n = 5) using flow cytometry. (**B)** We measured the expression of the degranulation marker CD107a in CTLs, CIK cells, and NK cells generated by mDCs against primary autologous myeloma cells. (**C)** The killing capacity of activated lymphocytes by mDCs against primary autologous myeloma cells was investigated using an LDH-release cytotoxicity assay without or with anti-MHC class I mAb (n = 3). The data indicate that CLTs, CIK cells, and NK cells generated by the IL-15 mDCs (6 days) and IL-15 mDCs (8 days) groups exhibited stronger cytotoxicity than those of the conventional mDCs group. Data are representative from three independent experiments. *P < 0.05; ** P < 0.01; *** P < 0.001 (One-way ANOVA, multiple comparisons test: Tukey for B. Multiple t tests – one per row for C).

To investigate the anti-myeloma effects of specific T cells and CIK cells generated by DCs loaded with gamma-irradiated autologous primary myeloma cells from patients, we performed a quantitative direct cytotoxic assay by measuring the levels of LDH, along with a CD107a degranulation assay. We analyzed the morphology of the patient primary myeloma cells for expression levels of MHC I, MICA, MICB, ULBP1, ULBP2/5/6, HLA-E, CD38 (n = 12), CD112, CD155, ICAM-1 and PD-L1 (n =5) using flow cytometry (Figure 7A). The data showed that primary myeloma cells expressed high levels of MHC I and MICA, indicating that T cells and CIK cells may mediate the cytotoxicity. In addition, from the GDC datasets, we found high levels of gene expression of MHC I, MICA and MICB (Supplemental Figure 10), which is identical to our surface markers data. The lymphocytes stimulated with IL-15 mDCs (6 days) and IL-15 mDCs (8 days) displayed significantly higher levels of LDH (Figure 7C) and higher expression of CD107a (n = 3) (Figure 7B) compared to the conventional mDCs group. With the addition of anti-MHC class I mAbs, levels of LDH decreased significantly in all three groups. However, the IL-15 mDCs (6 days) and IL-15 mDCs (8 days) groups still showed significantly higher levels of LDH compared to the conventional mDCs group (Figure 7C), indicating stronger activation of CIK cells and NK cells in the IL-15 mDCs (6 days) and IL-15 mDCs (8 days) groups compared to the conventional mDCs group. These results indicate that DCs generated with IL-15 significantly enhanced the cytotoxicity and function of lymphocytes, as indicated by the levels of LDH and CD107a expression against patient's autologous primary myeloma cells.

## Discussion

MM is characterized by immune suppression, such as dysfunction of effector lymphocytes, generation of immunosuppressive cells, and metabolites and cytokines that reduce anti-tumor immunity [23]. In addition, functional disability of DCs is frequently accompanied by cytokines and inhibitory proteins secreted by malignant plasma cells and the tumor microenvironment in MM patients [24]. These immune dysfunctions are correlated to the progression and relapse of MM, making MM difficult to cure. Therefore, cellular immunotherapy has attracted attention as a way to treat MM by activating, restoring, or augmenting immune function. DCs are a promising cellular immunotherapy for the treatment of MM because they can induce potent myeloma-specific CTLs without significant side effects. In our previous study, we generated potent DCs with a high migration capacity and performed a phase I/IIa clinical study for patients with relapsed or refractory MM [10]. DC vaccination did not result in any significant side effects, but the clinical efficacy was not satisfactory. Therefore, we are now focusing on developing more potent DCs.

There are several documents used only GM-CSF + IL-15 into DCs culture [14, 15], which result in high levels of IFN-γ but low levels of IL-12p70. Furthermore, the lack of IL-4 may result in the overgrowth of macrophage, and produce immature DCs that are unable to maintain an immature state, thus have decreased antigen-capturing capacity [11, 13, 16]. This study used culture method of GM-CSF + IL-15 + IL-4, which can overcome these shortages. In addition, this study was also the first to report the stimulation to CIK cells based on the high amount of IFN-γ from DCs. In this study, we cultured patient PBMCs with minimal supportive cytokines to mimic immunological reactions in MM patients. Also, we only use conventional maturating agents (LPS), to emphasize our study on the influence of IL-15 to dendritic cells culture, and make room for improvement in the future study by using stronger maturating agents on our novel IL-15 DCs. The data indicate that DC activation and stimulatory capacity regarding lymphocytes were remarkably enhanced by the addition of IL-15 during the period from monocytes to imDCs. Furthermore, multipotent IL-15 DCs that were cultured for a short duration (just 6 days) produced equal or better results compared to those cultured for 8 days. Thus, DC cultivation with IL-15 could be shortened to 6 days, reducing human resources, costs, and the risk of contamination. Some studies reported that monocyte-derived DCs can be generated after short-time cell culture (2-4 days) [14, 25], however data showed high level of monocytes marker CD14, which is not efficient in antigen presenting. The mechanism behind this is not fully understood; however, maybe due to a subpopulation of circulating monocytes represent a pool of standby precursor cells capable of rapid differentiation into mature DCs [26].

DCs from MM patients generated with IL-15 for 6 days showed higher expression of MHC I and MHC II, CD40, CD86, and migration receptor (CCR7) compared to conventional DCs. The most important finding concerning IL-15 mDCs (6 days) was the high amounts of IFN-γ and IL-12p70 secreted, which could further boost antigen presentation and cross-presentation, and thus enhance the expression of MHC class I and MHC class II. The secretion of cytokines IL-4 and IL-10 by IL-15 mDCs was also lower than that of conventional mDCs.

The polarization of allogeneic T cells was remarkably altered by IL-15 mDCs toward Th1. The crosstalk between DCs and allogeneic T cells occurred mainly via cytokine secretion [10, 17], implying that the high concentrations of IFN-γ and low concentrations of IL-4 and IL-10 caused by IL-15 mDCs promoted polarization of Th1 and inhibited polarization of Th2. IL-15 mDCs also showed higher stimulatory capacity regarding autologous T cells, a higher proportion of CD44^+^ and IFN-γ^+^ in CD4^+^ and CD8^+^ T cells, and higher cytotoxicity of T cells against cancer cell lines and patient autologous primary myeloma cells. Furthermore, IL-15 mDCs significantly inhibited regulatory T cells compared with controls.

Notably, IL-15 mDCs showed very good stimulatory capacity for autologous CIK cells, a higher proportion of CD44^+^, IFN-γ^+^, granzyme B^+^, and NKG2D in CIK cells, and a higher cytotoxicity of CIK cells against cancer cell lines and patient autologous primary myeloma cells. In previous clinical studies, CIK cells expanded with a high amount of IFN-γ, followed by supplement of IL-2 and CD3 antibody [27]. Incidentally, the IL-15 mDCs in the present study also secreted high amounts of IFN-γ, which may enhance CIK cell expansion. CIK cells can display potent cytolytic activities, via not only MHC-restricted specific recognition but also TCR-independent NK-like function [28]. Recently, the combination of DCs and CIK cells for cancer immunotherapy has been an intense focus of study [29, 30]. Because the IL-15 mDCs in the present study showed very good stimulatory capacity for CIK cells, they could be used as an alternative to conventional DCs in combination with CIK cells. In addition, IL-15 mDCs dominantly enhanced the activation and cytotoxicity of autologous NK cells. The enhancement of NK cells may be caused by the short exposure to membrane binding IL-15 in IL-15 DCs [31]. Despite the higher production of IFN-γ of CIK cells in the IL-15 mDCs (6 days) group compared to IL-15 mDCs (8 days) group, we did not observe a significant different with CD107a degranulation assay against both cancer cells line and autologous primary myeloma cells. On the other hand, with the higher production of granzyme B of NK cells in the IL-15 mDCs (6 days group), associated with enhance cytotoxicity with CD107a degranulation assay against K562 cancer cells compared to other groups. Enhancement of NK cells in DC-based vaccines is important because activated NK cells can help T cells to eradicate cancer cells that lose expression of MHC class I. Additionally, in vivo tumor-infiltrating NK cells can recruit more DCs through the secretion of CCL5 and XC-chemokine ligand 1 (XCL1), along with enhanced DC development and proliferation through secretion of FMS-related tyrosine kinase 3 ligand (FLT3L) [7]. Furthermore, the high expression level on CD40L in activated CIK cells and T cells [32, 33], may give positive feedback to mature DCs, and increase the secretion levels of IL-12 and IFN-γ; via that enhance the proliferation and function of T cells, CIK cells and NK cells even further.

Our results indicate that the DCs generated with IL-15 from MM patients had outstanding activation capacities for autologous T cells, CIK cells, and NK cells. Our results (Figure 7A), together with the gene-expression data from MM samples collected by the Multiple Myeloma Research Foundation (MMRF) in the Genomic Data Commons (GDC) data portal [22], indicate that most myeloma cells show high expression of HLA-ABC, MICA, and MICB, which are suitable for T cell and CIK cell immunotherapy (Figure 7A and Supplemental Figure 10A). Autologous CIK immunotherapy can also address the high expression of inhibitory HLA-E on myeloma cells, due to the absence of expression of CD94/NKG2A on CIK cells [27]. Our data show that the high expression of CD38 on myeloma cells make it suitable for using immunotherapy in combination with monoclonal antibody directed against CD38, such as Daratumumab. Our data also show that IL-15 mDCs, especially IL-15 mDCs (6 days), can inhibit regulatory T cells, which can further enhance the overall survival rate of MM patients (Supplemental Figure 10B).

The limitation of this study is that we did not use a mouse model of MM, which is a potent tool for studying MM progression and development. In the future, we plan to develop intravenous and subcutaneous myeloma mouse models to mimic circulating myeloma and extramedullary plasmacytoma, and to further expand our experiments in vivo.

In conclusion, to our knowledge, this is the first study about ex vivo culturing DCs by addition of IL-15 in MM. We generated novel multipotent DCs by adding IL-15 to cells from MM patients and incubating them for 6 days. The resulting IL-15 DCs (6 days) exhibited outstanding activation of autologous T cells, CIK cells, and NK cells, and demonstrated strong cytolytic activity against both myeloma cell lines and patient autologous primary myeloma cells. Our findings may serve as a cornerstone of DC-based immunotherapy with novel multipotent IL-15 mDCs in the field of cancer immunotherapy.

## Funding

This work was supported by the 10.13039/501100003725National Research Foundation of Korea (NRF) grant funded by the Korea government (NRF-2020R1A5A2031185, NRF-2020R1A2C2010098). This study was also supported by a grant from the Bio & Medical Technology Development Program of the 10.13039/501100001321National Research Foundation (NRF), funded by the Korean government (MSIT) (NRF-2020M3A9G3080330).

## Declaration of Competing Interest

The authors declare no conflicts of interest.

## Appendix Supplementary materials

**Supplemental Figure 1. Characterization of GM-CSF + IL-4 + IL-15 mature (m)DCs.** We analyzed the DC phenotype of imDCs (serve as negative control) and mDCs for expression levels of CCR7, MHC I and II, CD80, CD86 using flow cytometry at Day 8. **(A)** The data was showed via representative histogram (sample in shaded, compared to isotype control in black line) and **(B)** the MFI ratios (MFI of samples/MFI of isotype controls) of each sample are shown as bar graphs. All mDCs groups showed increased expression levels of CCR7, MHC I and II, CD80, CD86 compared to their imDCs counterparts. However, GM-CSF + IL-15 mDCs group did not show significant different in expression levels of CCR7 compared to GM-CSF + IL-15 imDCs. GM-CSF + IL-4 + IL-15 mDCs group showed increased in expression of CD80 and CD86 compared to GM-CSF + IL-15 mDCs group and GM-CSF + IL-4 mDCs group. We analyzed **(C)** CD11c and **(D)** CD14 expression on GM-CSF + IL-4 + IL-15 imDCs at day 4, data showed via representative dot plot. Data are representative from three independent experiments (n =3). *P < 0.05; ** P < 0.01; *** P < 0.001; **** P < 0.0001; ns: no significant difference (One-way ANOVA, multiple comparisons test: Tukey). Image, image 1

Supplemental Figure 2. GM-CSF + IL-4 + IL-15 mDCs produced stronger cytokines profile toward enhanced activation of immune cells. ELISA results from supernatants of imDCs and mDCs cultured with CD40L-transfected J558 cells show that GM-CSF + IL-4 + IL-15 mDCs groups produced the highest level of (A) IFN-γ and lower levels of (B) IL-4 and (C) IL-10 compared to the GM-CSF + IL-4 (conventional mDCs) group. Levels of (D) IL-12p70 in GM-CSF + IL-4 + IL-15 mDCs group was higher compared to GM-CSF + IL-15 mDCs group. GM-CSF + IL-15 mDCs group did not show significant different in levels of IL-12p70 compared to GM-CSF + IL-15 imDCs group. Data are representative from three independent experiments (n =3). ** P < 0.01; *** P < 0.001; **** P < 0.0001; ns: no significant difference (One-way ANOVA, multiple comparisons test: Tukey). Image, image 2

**Supplemental Figure 3. HLA-A2 expression on MM patient, ARH77, IM9, and K562 cancer cells.** The expression of HLA-A2 in lymphocytes of MM patients was measured by flow cytometry, immediately after collected PBMC. **(A)** All patient's cells expressed HLA-A2 (n = 10). **(B)** ARH77 and IM9 cancer cells expressed HLA-A2, while K562 cells did not. **(C)** We measured the antigen uptake capacity of mDCs against gamma-irradiated ARH77 cancer cells using flow cytometry (n = 3). Data are representative from three independent experiments. *P < 0.05 (One-way ANOVA, multiple comparisons test: Tukey). Image, image 3

**Supplemental Figure 4. Characterization of DCs via representative histogram.** We analyzed the DC phenotypes to assess the expression levels of CD3, CD56, CD40, CD80, CD83, CD86, CCR7, MHC I, and MHC II using flow cytometry. Representative histogram figures include marker expression levels (shaded) compared to the isotype controls (black line). Data are representative from ten independent experiments. Image, image 4

Supplemental Figure 5. Flow cytometry gating strategies in autologous effector lymphocytes activated by mDCs. We performed several flow cytometry gating strategies in autologous effector lymphocytes. Image, image 5

Supplemental Figure 6. IFN-γ expression on autologous CD4^+^ T cells, CD8^+^ T cells, CIK cells, and NK cells activated by mDCs. We measured the expression of intracellular IFN-γ by flow cytometry for (A) CD4^+^ T cells, (B) CD8^+^ T cells, (C) CIK cells, and (D) NK cells. Lymphocytes stimulated by IL-15 mDCs (6 days) showed higher expression of IFN-γ on lymphocytes compared to those of conventional mDCs. Data are representative from three independent experiments (n = 3). *P < 0.05; ** P < 0.01; *** P < 0.001; **** P < 0.0001 (One-way ANOVA, multiple comparisons test: Tukey). Image, image 6

**Supplemental Figure 7. Granzyme B expression on autologous CD8^+^ T cells, CIK cells, and NK cells activated by mDCs.** We measured the expression of intracellular granzyme B by flow cytometry on **(A)** CD8^+^ T cells, **(B)** NK cells, and **(C)** CIK cells. The IL-15 mDCs (6 days) group showed the highest expression of granzyme B on NK cells and CIK cells compared to the other groups. Data are representative from three independent experiments (n = 3). *P < 0.05; ** P < 0.01; *** P < 0.001 (One-way ANOVA, multiple comparisons test: Tukey). Image, image 7

**Supplemental Figure 8. NKG2D expression on autologous CIK cells and NK cells activated by mDCs.** We measured the expression of NKG2D by flow cytometry on **(A)** NK cells and **(B)** CIK cells. IL-15 mDCs (6 days) showed higher expression of NKG2D on NK cells and CIK cells compared to the conventional mDCs group. Data are representative from three independent experiments (n = 3). *P < 0.05; ** P < 0.01; *** P < 0.001 (One-way ANOVA, multiple comparisons test: Tukey). Image, image 8

**Supplemental Figure 9. tSNE of every activated population in lymphocytes.** Representative t-distributed stochastic neighbor embedding (tSNE) in lymphocyte/live cell populations (total cells = 10,000 cells) of activated CD4^+^ T cells, CD8^+^ T cells, CIK cells, and NK cells. Image, image 9

**Supplemental Figure 10. HLA-ABC, MICA, MICB, ULBP1, ULBP2, HLA-E, and FOXP3 expression on MM patient cells in GDC datasets.** We assessed the gene expression of cell samples from MM patients from the MMRF-COMMPASS GDC datasets in the Xena browser. **(A)** The gene expression of HLA-A, HLA-B, HLA-C, MICA, MICB, ULBP1, ULBP2, and HLA-E on MM samples, and (**B)** the survival curve of two different patient populations based on high and low FOXP3 expression. We found high expression of MHC class I, MICA, and MICB on myeloma cells, which are suitable for autologous T cell and CIK cell therapy. Image, image 10
