# Supplementary figures and images for: Novel IL-15 dendritic cells have a potent immunomodulatory effect in immunotherapy of multiple myeloma
Source: Transl Oncol. 2022 Apr 9;20:101413. doi: 10.1016/j.tranon.2022.101413 (PMC9006865; doi:10.1016/j.tranon.2022.101413)

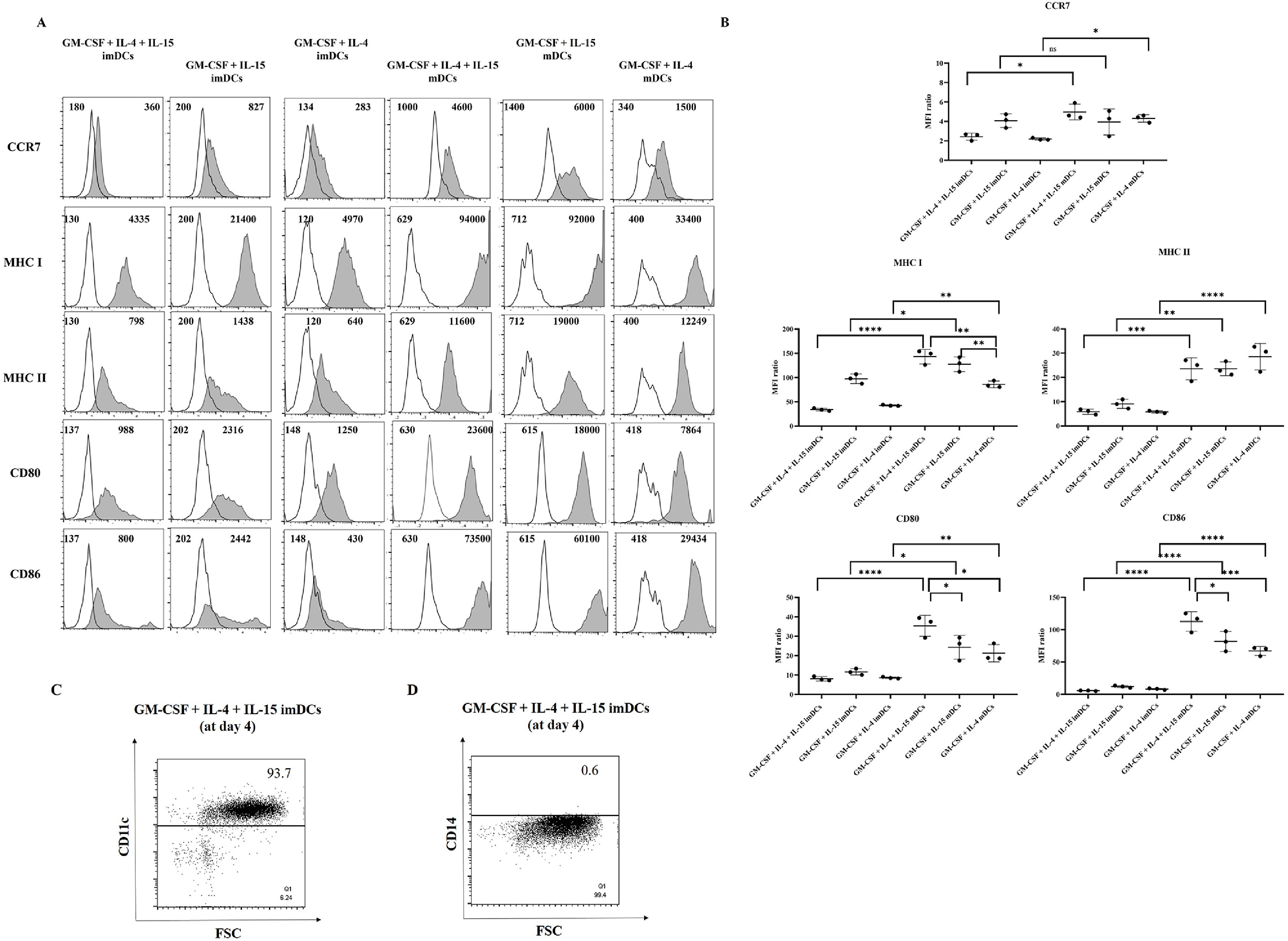

Supplement: Supplementary file 1 — Supplemental Figure 1. Characterization of GM-CSF + IL-4 + IL-15 mature (m)DCs. We analyzed the DC phenotype of imDCs (serve as negative control) and mDCs for expression levels of CCR7, MHC I and II, CD80, CD86 using flow cytometry at Day 8. (A) The data was showed via representative histogram (sample in shaded, compared to isotype control in black line) and (B) the MFI ratios (MFI of samples/MFI of isotype controls) of each sample are shown as bar graphs. All mDCs groups showed increased expression levels of CCR7, MHC I and II, CD80, CD86 compared to their imDCs counterparts. However, GM-CSF + IL-15 mDCs group did not show significant different in expression levels of CCR7 compared to GM-CSF + IL-15 imDCs. GM-CSF + IL-4 + IL-15 mDCs group showed increased in expression of CD80 and CD86 compared to GM-CSF + IL-15 mDCs group and GM-CSF + IL-4 mDCs group. We analyzed (C) CD11c and (D) CD14 expression on GM-CSF + IL-4 + IL-15 imDCs at day 4, data showed via representative dot plot. Data are representative from three independent experiments (n =3). *P < 0.05; ** P < 0.01; *** P < 0.001; **** P < 0.0001; ns: no significant difference (One-way ANOVA, multiple comparisons test: Tukey). [file mmc1.jpg]

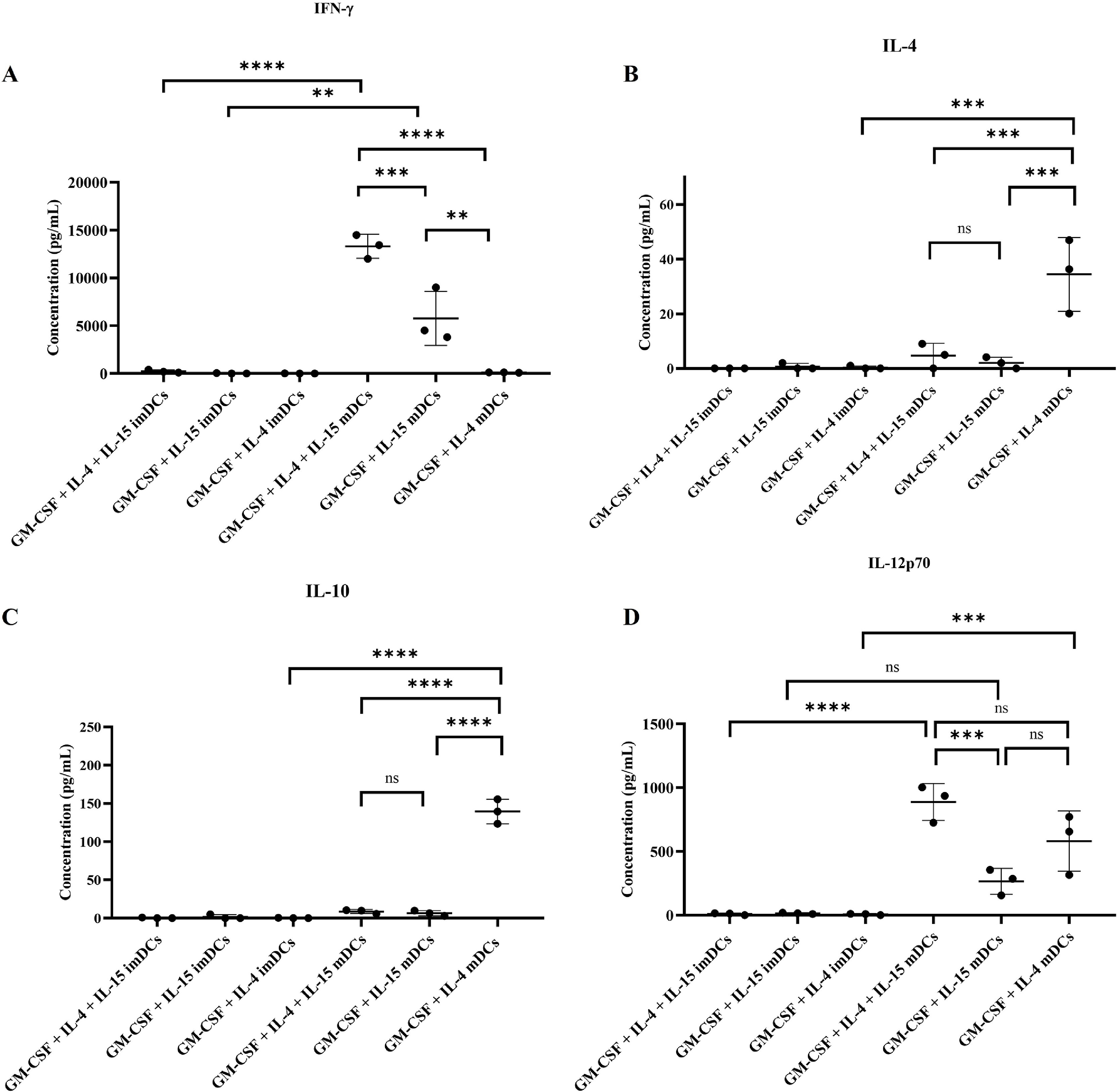

Supplement: Supplementary file 2 — Supplemental Figure 2. GM-CSF + IL-4 + IL-15 mDCs produced stronger cytokines profile toward enhanced activation of immune cells. ELISA results from supernatants of imDCs and mDCs cultured with CD40L-transfected J558 cells show that GM-CSF + IL-4 + IL-15 mDCs groups produced the highest level of (A) IFN-γ and lower levels of (B) IL-4 and (C) IL-10 compared to the GM-CSF + IL-4 (conventional mDCs) group. Levels of (D) IL-12p70 in GM-CSF + IL-4 + IL-15 mDCs group was higher compared to GM-CSF + IL-15 mDCs group. GM-CSF + IL-15 mDCs group did not show significant different in levels of IL-12p70 compared to GM-CSF + IL-15 imDCs group. Data are representative from three independent experiments (n =3). ** P < 0.01; *** P < 0.001; **** P < 0.0001; ns: no significant difference (One-way ANOVA, multiple comparisons test: Tukey). [file mmc2.jpg]

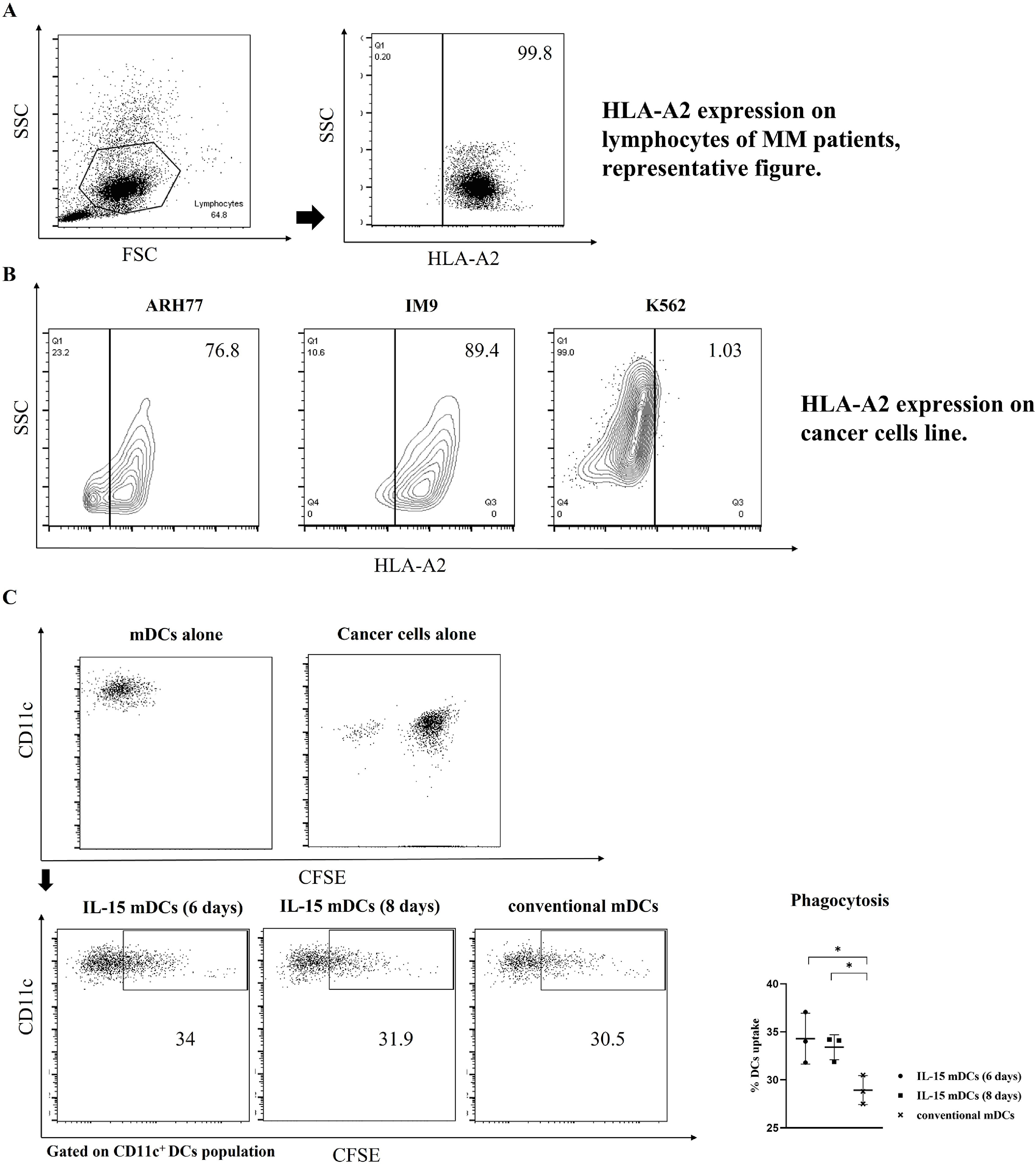

Supplement: Supplementary file 3 — Supplemental Figure 3. HLA-A2 expression on MM patient, ARH77, IM9, and K562 cancer cells. The expression of HLA-A2 in lymphocytes of MM patients was measured by flow cytometry, immediately after collected PBMC. (A) All patient's cells expressed HLA-A2 (n = 10). (B) ARH77 and IM9 cancer cells expressed HLA-A2, while K562 cells did not. (C) We measured the antigen uptake capacity of mDCs against gamma-irradiated ARH77 cancer cells using flow cytometry (n = 3). Data are representative from three independent experiments. *P < 0.05 (One-way ANOVA, multiple comparisons test: Tukey). [file mmc3.jpg]

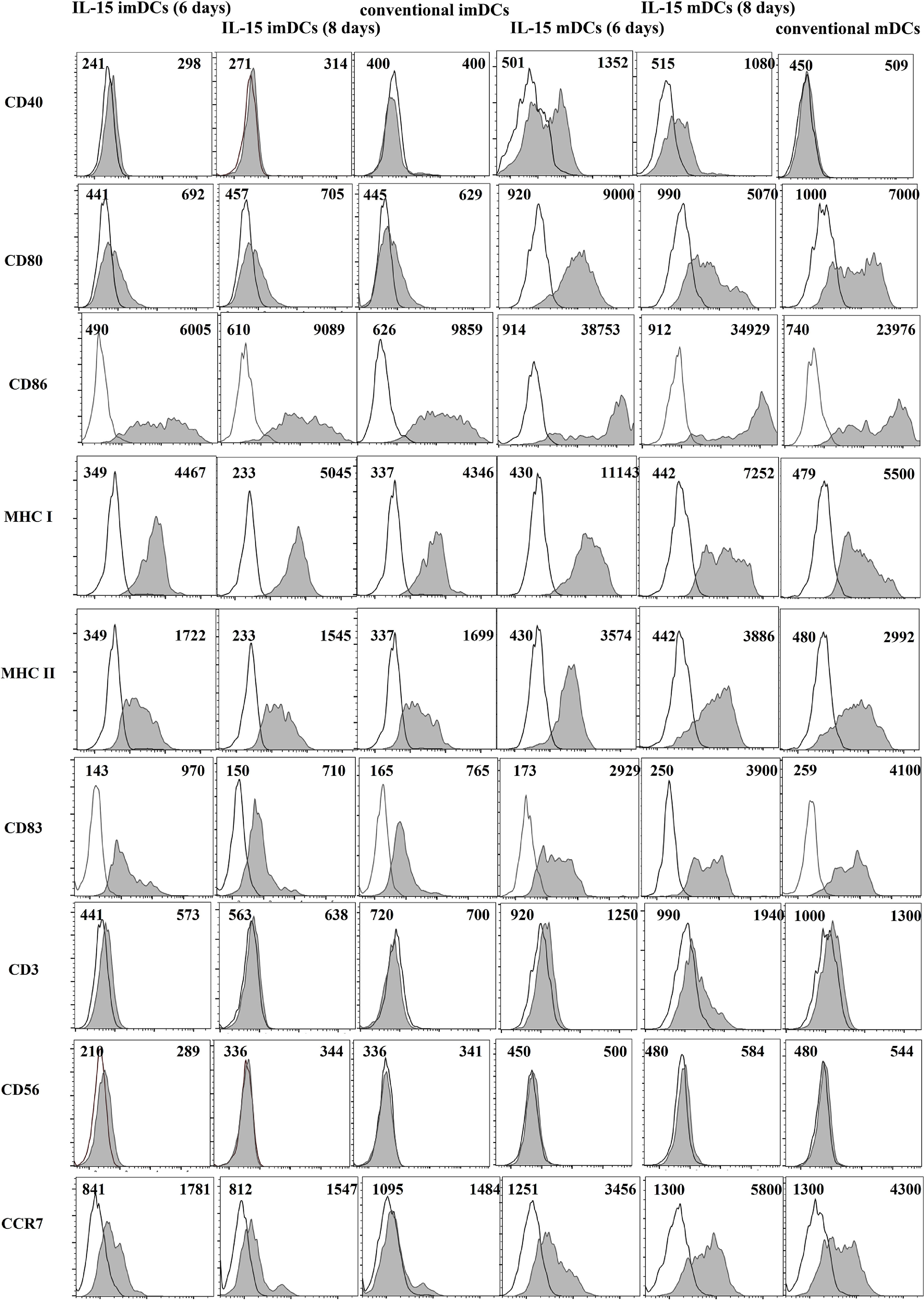

Supplement: Supplementary file 4 — Supplemental Figure 4. Characterization of DCs via representative histogram. We analyzed the DC phenotypes to assess the expression levels of CD3, CD56, CD40, CD80, CD83, CD86, CCR7, MHC I, and MHC II using flow cytometry. Representative histogram figures include marker expression levels (shaded) compared to the isotype controls (black line). Data are representative from ten independent experiments. [file mmc4.jpg]

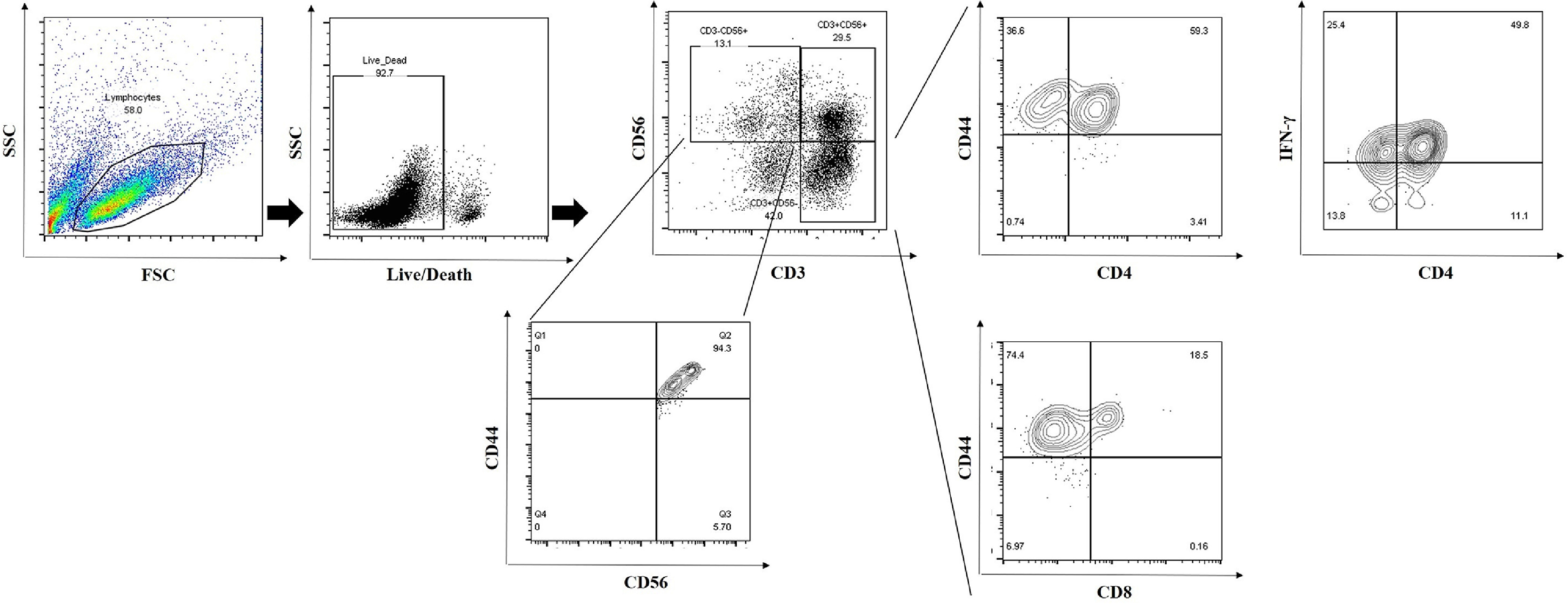

Supplement: Supplementary file 5 — Supplemental Figure 5. Flow cytometry gating strategies in autologous effector lymphocytes activated by mDCs. We performed several flow cytometry gating strategies in autologous effector lymphocytes. [file mmc5.jpg]

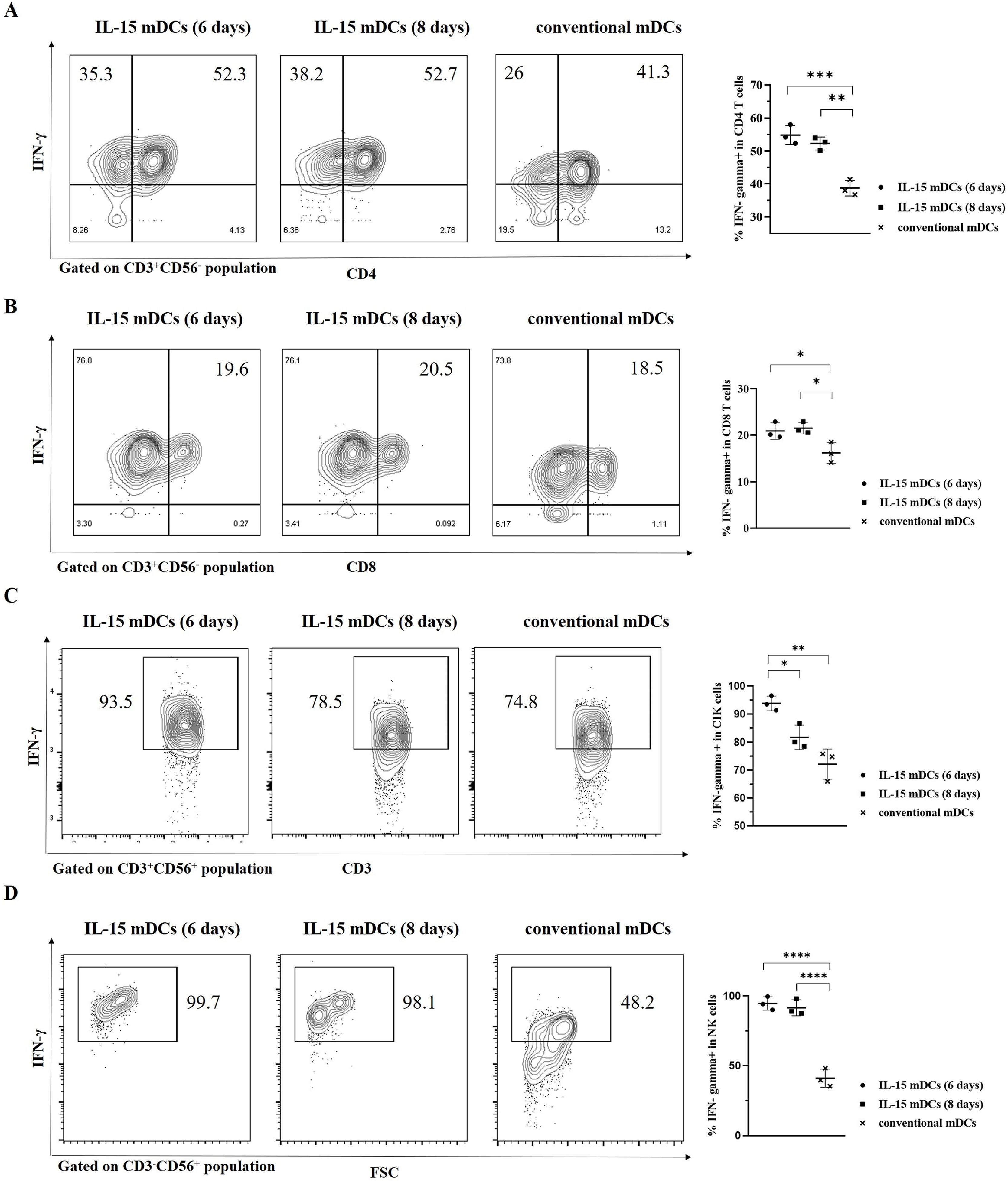

Supplement: Supplementary file 6 — Supplemental Figure 6. IFN-γ expression on autologous CD4+ T cells, CD8+ T cells, CIK cells, and NK cells activated by mDCs. We measured the expression of intracellular IFN-γ by flow cytometry for (A) CD4+ T cells, (B) CD8+ T cells, (C) CIK cells, and (D) NK cells. Lymphocytes stimulated by IL-15 mDCs (6 days) showed higher expression of IFN-γ on lymphocytes compared to those of conventional mDCs. Data are representative from three independent experiments (n = 3). *P < 0.05; ** P < 0.01; *** P < 0.001; **** P < 0.0001 (One-way ANOVA, multiple comparisons test: Tukey). [file mmc6.jpg]

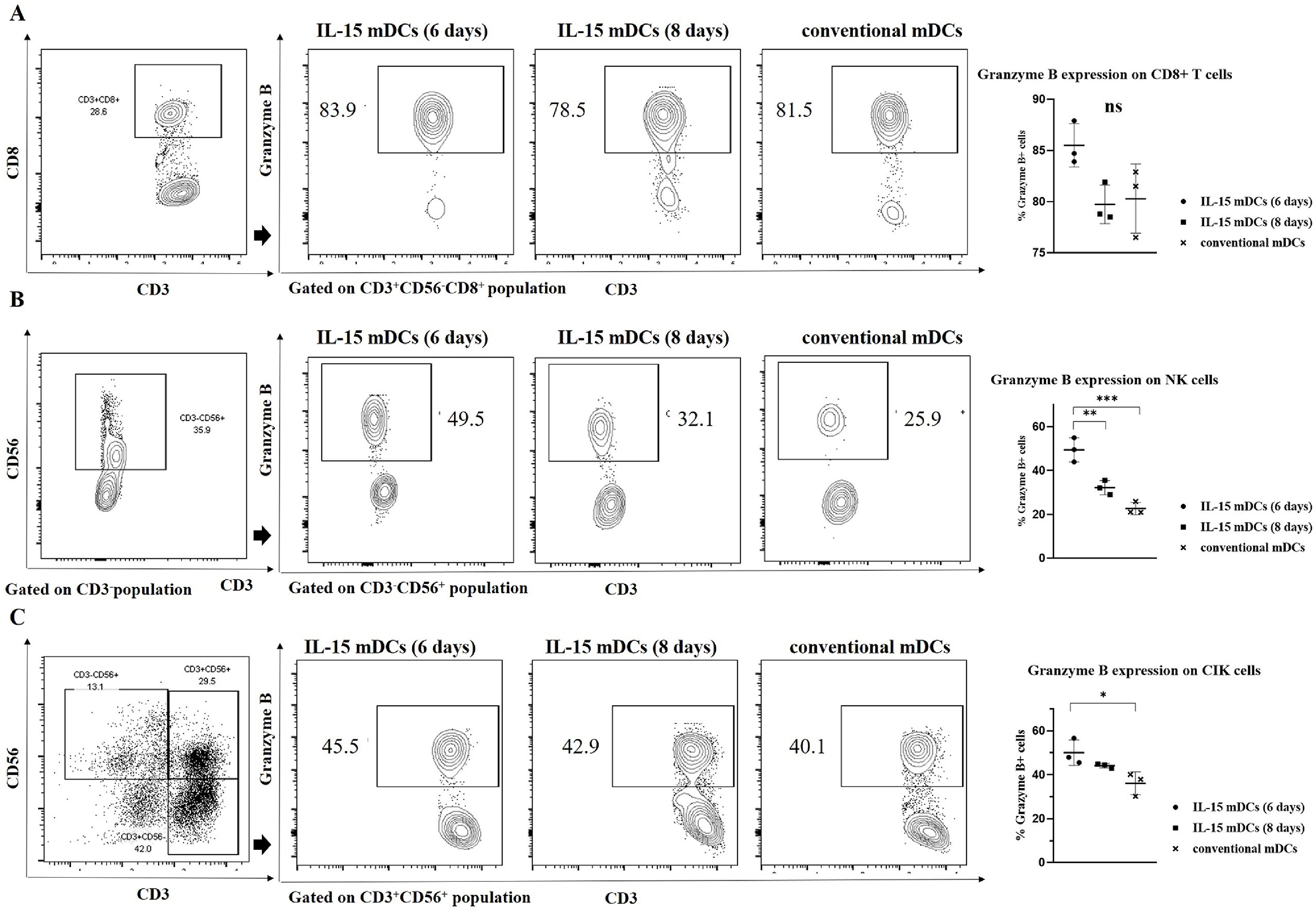

Supplement: Supplementary file 7 — Supplemental Figure 7. Granzyme B expression on autologous CD8+ T cells, CIK cells, and NK cells activated by mDCs. We measured the expression of intracellular granzyme B by flow cytometry on (A) CD8+ T cells, (B) NK cells, and (C) CIK cells. The IL-15 mDCs (6 days) group showed the highest expression of granzyme B on NK cells and CIK cells compared to the other groups. Data are representative from three independent experiments (n = 3). *P < 0.05; ** P < 0.01; *** P < 0.001 (One-way ANOVA, multiple comparisons test: Tukey). [file mmc7.jpg]

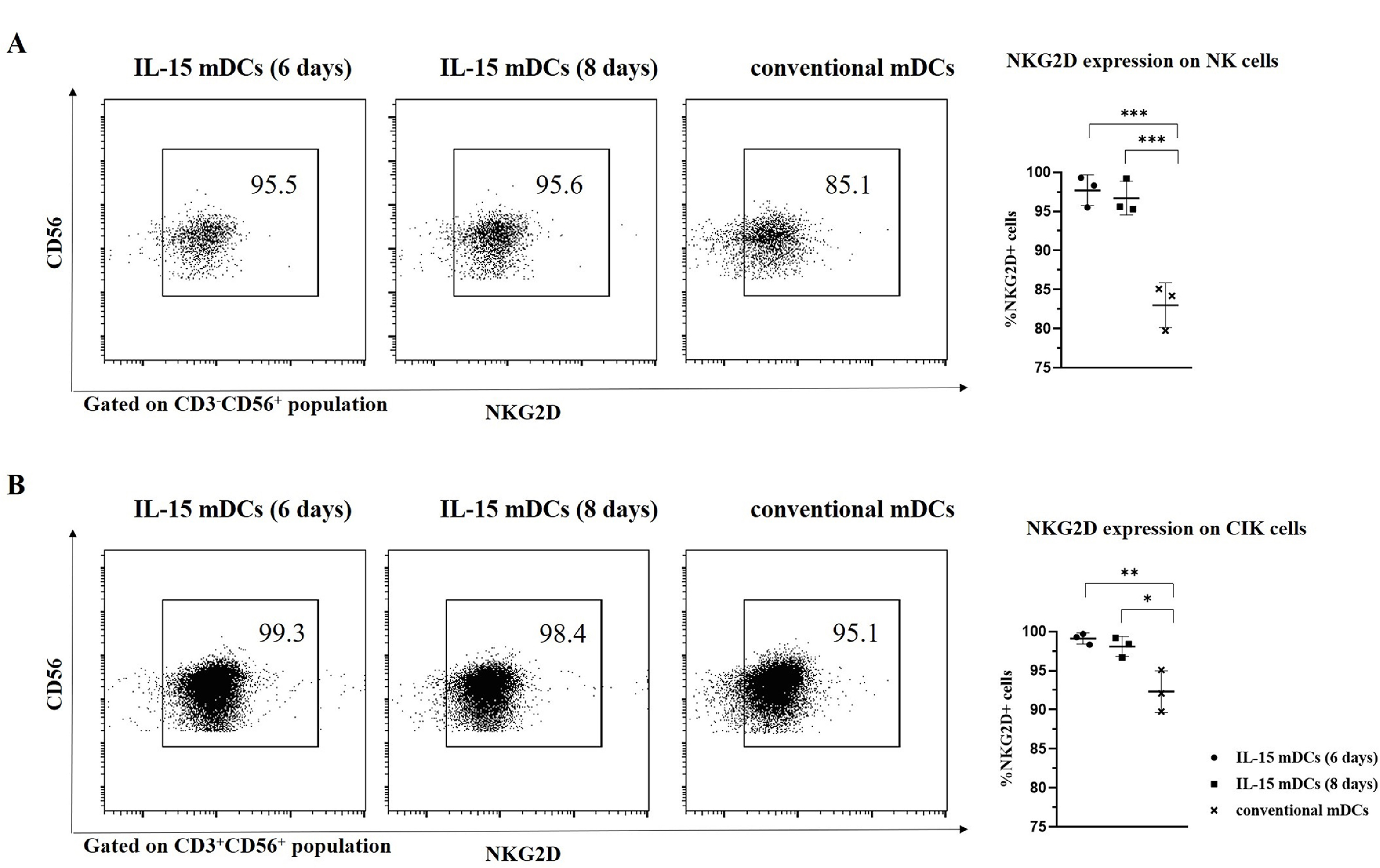

Supplement: Supplementary file 8 — Supplemental Figure 8. NKG2D expression on autologous CIK cells and NK cells activated by mDCs. We measured the expression of NKG2D by flow cytometry on (A) NK cells and (B) CIK cells. IL-15 mDCs (6 days) showed higher expression of NKG2D on NK cells and CIK cells compared to the conventional mDCs group. Data are representative from three independent experiments (n = 3). *P < 0.05; ** P < 0.01; *** P < 0.001 (One-way ANOVA, multiple comparisons test: Tukey). [file mmc8.jpg]

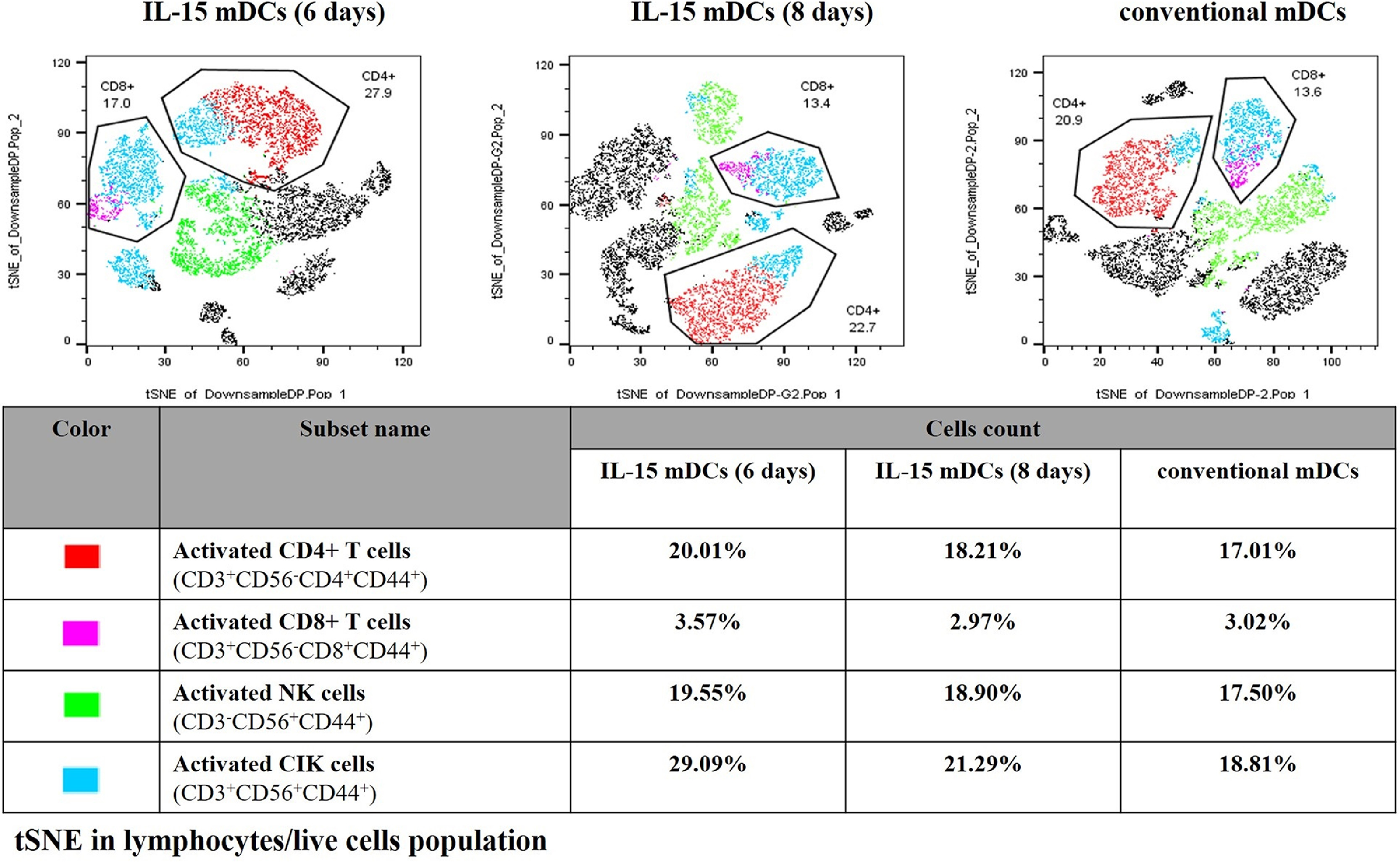

Supplement: Supplementary file 9 — Supplemental Figure 9. tSNE of every activated population in lymphocytes. Representative t-distributed stochastic neighbor embedding (tSNE) in lymphocyte/live cell populations (total cells = 10,000 cells) of activated CD4+ T cells, CD8+ T cells, CIK cells, and NK cells. [file mmc9.jpg]

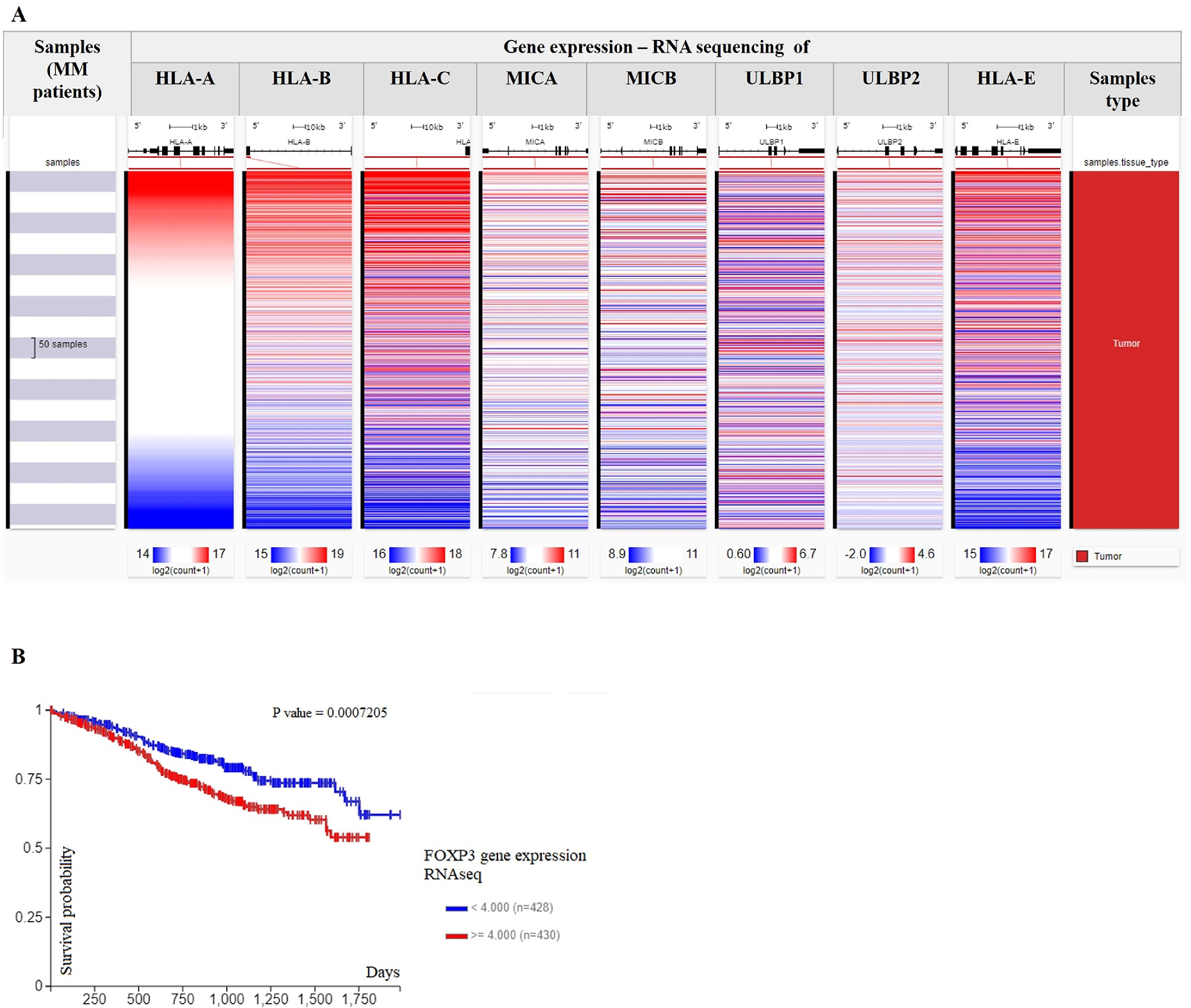

Supplement: Supplementary file 10 — Supplemental Figure 10. HLA-ABC, MICA, MICB, ULBP1, ULBP2, HLA-E, and FOXP3 expression on MM patient cells in GDC datasets. We assessed the gene expression of cell samples from MM patients from the MMRF-COMMPASS GDC datasets in the Xena browser. (A) The gene expression of HLA-A, HLA-B, HLA-C, MICA, MICB, ULBP1, ULBP2, and HLA-E on MM samples, and (B) the survival curve of two different patient populations based on high and low FOXP3 expression. We found high expression of MHC class I, MICA, and MICB on myeloma cells, which are suitable for autologous T cell and CIK cell therapy. [file mmc10.jpg]
